# The Biological Significance of Targeting Acetylation-Mediated Gene Regulation for Designing New Mechanistic Tools and Potential Therapeutics

**DOI:** 10.3390/biom11030455

**Published:** 2021-03-18

**Authors:** Chenise O’Garro, Loveth Igbineweka, Zonaira Ali, Mihaly Mezei, Shiraz Mujtaba

**Affiliations:** 1 Department of Biology, Medgar Evers College, City University of New York, Brooklyn, NY 11225, USA; chenise.ogarro@student.mec.cuny.edu (C.O.); L.Igbineweka@mec.cuny.edu (L.I.); zonaira.ali@student.mec.cuny.edu (Z.A.); 2 Department of Pharmaceutical Sciences, Icahn School of Medicine at Mount Sinai, New York, NY 10029, USA; mihaly.mezei@mssm.edu

**Keywords:** epigenetic modifications, acetylation, lysine acetyltransferases, gene regulation, molecular interactions and biological outcomes

## Abstract

The molecular interplay between nucleosomal packaging and the chromatin landscape regulates the transcriptional programming and biological outcomes of downstream genes. An array of epigenetic modifications plays a pivotal role in shaping the chromatin architecture, which controls DNA access to the transcriptional machinery. Acetylation of the amino acid lysine is a widespread epigenetic modification that serves as a marker for gene activation, which intertwines the maintenance of cellular homeostasis and the regulation of signaling during stress. The biochemical horizon of acetylation ranges from orchestrating the stability and cellular localization of proteins that engage in the cell cycle to DNA repair and metabolism. Furthermore, lysine acetyltransferases (KATs) modulate the functions of transcription factors that govern cellular response to microbial infections, genotoxic stress, and inflammation. Due to their central role in many biological processes, mutations in KATs cause developmental and intellectual challenges and metabolic disorders. Despite the availability of tools for detecting acetylation, the mechanistic knowledge of acetylation-mediated cellular processes remains limited. This review aims to integrate molecular and structural bases of KAT functions, which would help design highly selective tools for understanding the biology of KATs toward developing new disease treatments.

## 1. Introduction

Cells have extraordinary potential to calibrate appropriate responses to the everchanging environment for maintaining tissue integrity and physiological balance. One of the longstanding biomedical research goals has been to fully reveal the molecular mechanisms that direct human development, maintain homeostasis, and calculate the intensity of cellular responses to internal and extracellular changes [1,2]. These changes in the cellular milieu stem from hormonal regulation, infections, immune activation, metabolic imbalances, and genotoxic stresses [3]. Intriguingly, despite their identical genomes, it is unclear how cells within the embryonic layers acquire distinct and tissue-specific traits. The multilayered molecular mechanisms that determine a cell’s fate remain to be fully understood. Mounting evidence confirms that the epigenetic mechanisms controlling the gene expression system play a vital role in modulating cellular functions in response to environmental changes [4,5,6,7,8]. These epigenetic mechanisms lie at the center of regulating stress responses, metabolic pathways, and diseases (e.g., neurological disorders, chronic inflammation, microbial infections, and cancers) [9,10,11]. The posttranslational modifications, molecular interactions, and noncoding RNAs comprise major epigenetic mechanisms that regulate the outcomes of gene transcription [12,13,14,15]. Additionally, posttranslational modification-mediated nucleosomal remodeling controls the transcriptional machinery’s recruitment to the gene promoters, intragenic regions, and enhancers [5,16]. A detailed mechanistic analysis of epigenetic pathways that govern gene functions and cellular responses will help to understand normal and pathological situations and aid in developing therapeutic modalities. However, developing a highly selective tool is vital to fully understand the molecular basis of epigenetic mechanisms.

Nucleosomes are composed of DNA and octameric histone proteins. They undergo a wide array of chemical modifications triggered by upstream signals, which enable the modulation of downstream target genes [16]. As compared to the methylation of adenine and cytosine nucleotides—a major DNA modification—proteins undergo posttranslational modifications on residues including serine, threonine, tyrosine, lysine, and arginine [17,18,19]. These epigenetic modifications include, but are not limited to, phosphorylation, acetylation, methylation, ubiquitination, and sumoylation; which are mediated by kinases, lysine acetyltransferases (KATs), lysine methyltransferases, ubiquitinases, and sumoylases, respectively [20,21,22]. Serendipitously, most epigenetic modifications are reversible, thereby facilitating modification-dependent and modification-independent molecular interactions [20,21,22]. Reversal of epigenetic modifications is mediated by phosphatases, lysine deacetylases (KDACs), demethylases, and deubiquitinases [23,24,25,26]. The dynamics—catalyzing epigenetic modifications followed by swift removal—broaden the plasticity in cellular responses. During traumatic brain injury, the molecular interplay of KAT2B, KAT3A, and Nicotinamide adenine dinucleotide (NAD)-dependent deacetylase Sirtuin 1 regulates the expression of genes regulated by a hypoxic environment [27]. Interestingly, the interactions between KAT3B and NAD-dependent Sirtuin 1 also regulate metabolic processes [28]. Collectively, these molecular events serve as a target to manipulate cell fate for changing disease outcomes.

Depending upon their roles in regulating transcriptional outcomes, chromatin-associated proteins can be defined as transcriptional cofactors, further characterized as coactivators or corepressors [29,30,31]. The genetic mutations within transcriptional coactivators lead to developmental disorders, long-term intellectual challenges, and increased susceptibility to life-threatening diseases [32]. Furthermore, dysregulated coactivator functions perturb the stoichiometric balance with transcription factors, which adversely affects downstream gene functions, leading to disease outcomes [29,30,31]. For instance, deregulated dynamics of KATs or KDACs on the androgen receptor (AR) target gene promoters escalate anti-androgen resistance, which leads to the growth and metastasis of prostate cancer (PCa) cells [33,34,35]. These data implicate the translational significance of coactivators, which have naturally become the target for a generation of new medicines [9,36]. Gene manipulation strategies may not be widely useful, for at least two reasons: first, coactivators are multidomain proteins that mediate several functions, and second, gene editing can generate unintended secondary mutations and off-target effects [37]. Small molecule-mediated perturbations of endogenous coactivator functions can elucidate the mechanistic underpinnings of crucial mechanisms that will help develop high-affinity therapeutic modalities. Although the role of phosphorylation was discovered much earlier in bridging cellular signaling networks, lysine acetylation was noted for the first time in 1964 [38]. This review aims to integrate the biochemical, molecular, and structural bases of KAT functions, which would increase our understanding of KAT-directed biology and its involvement in disease pathogenesis.

## 2. The Biochemistry and Epigenetics of Lysine Acetylation on Chromatin

**Figure 1:**
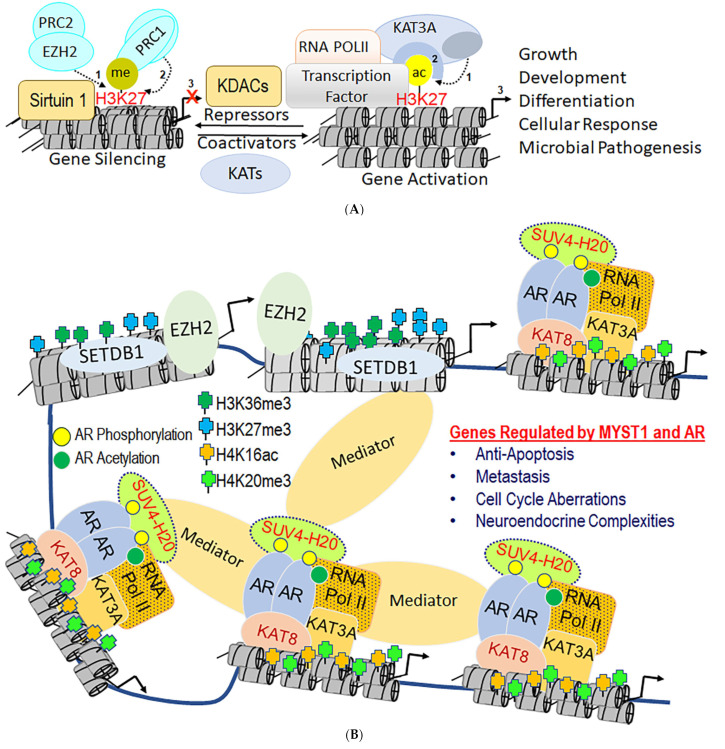
(**A**) Transcriptional coactivator (KAT)- and corepressor (KDAC)-mediated mutually exclusive epigenetic modifications on the chromatin landscape. H3K27 site can be methylated (me) by lysine methyltransferase (EZH2) and acetylated (ac) by KAT3A/-3B. While methylation of H3K27 leads to gene silencing, acetylation of H3K27 causes gene activation. EZH2 is a part of the polycomb repressor complex (PRC)-2, which enzymatically methylates H3K27 (1), and subsequently recruits methyl-lysine-binding chromodomain-containing proteins (which are a part of PRC-1). Similarly, acetylation of lysine residues by KATs (1) is followed by the recruitment of bromodomain on the acetylated lysine sites (2). Additionally, acetylated lysine residues can also recruit KDACs (like NAD-dependent Sirtuin 1) which deacetylate and cooperate with PRC complexes. On the one hand, these epigenetic modifications play a key role in growth, development, and differentiation; on the other hand, they can cause developmental disorders and cancers. (**B**) Global regulation of the human epigenome by lysine acetylation and methylation on chromatin has the potential to govern cellular responses by activation or silencing of individual and long-distance genes. Androgen Receptor (AR) is a ligand-binding transcription factor that undergoes phosphorylation (yellow balls) and acetylation (green balls) upon binding to the hormone testosterone. In normal situations, this would lead to the development of the prostate and secondary male characteristics. However, dysregulated dynamics of AR and coactivator functions have the potential to cause prostate cancer. In prostate cancer cells, coactivators KAT3A and KAT8 can acetylate AR and the chromatin of the target genes to induce cell proliferation and metastasis. Furthermore, the trimethylation of H3K27 and H4K20 sites by EZH2 and SUV4-20H2 creates a microenvironment that cooperates with KAT3-A and KAT8 to resist antiandrogen therapy. One of the roles of mediators in transcription is to serve as the chromatin organizers.

Acetylation of cellular proteins involves the KAT-mediated covalent linkage of the acetyl moiety from acetyl-CoA to the ε-N on the side chain of a lysine residue to form ε-N-acetyllysine [39]. An acetylated lysine moiety then becomes a pivotal hub for recruiting bromodomain-containing proteins or KDACs that trigger a wide range of biochemical activities and molecular interactions (Figure 1A,B) [40,41]. The association between acetylation of histone proteins and transcriptionally active regions has been experimentally established using deoxyribonuclease-1 sensitivity and chromatin immunoprecipitation (ChIP) assays [42]. Genome-wide ChIP-on-ChIP data have revealed that most KATs function as coactivators—cooperating with transcription factors, general transcription machinery, and RNA polymerase II to express target genes [43]. Furthermore, site-specific acetylation on a histone protein neutralizes the positive charge of the lysine residues, inducing chromatin remodeling to facilitate DNA access by transcriptional machinery [39,44,45,46]. In addition to chromatin, KAT-mediated acetylation modulates the functions of transcription factors by enhancing their stability and transcriptional activities (Figure 1A,B) [47]. The KAT2A/-2B and KAT5-mediated acetylation and molecular interactions with KAT3A/-3B increase the stability of the c-Myc protein [48]. Taken together, although we understand the role of acetylation in the regulation of protein-coding genes, the role of acetylation remains lesser known in the expression of noncoding genes.

**Figure 2:**
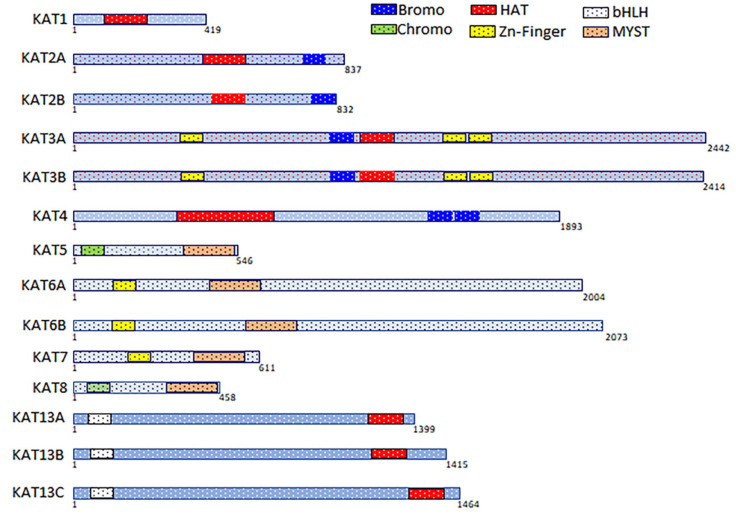
The domain composition and organization of KATs present in humans. KATs are multidomain proteins that perform more than one function. For instance, KAT2A/-2B, KAT3A/-3B, and KAT4 have a bromodomain, an acetyl-lysine binding module, whereas KAT8 has a MYST domain and a chromodomain.

Across the genome, there appear to be at least 20 KATs located on different chromosomes. They are composed of several domains, including bromodomain, chromodomain, one or more zinc fingers, plant homeodomain, MOZ, Ybf2 (Sas3), Sas2, and Tip60 (MYST; Figure 2) [20,39]. However, only 13 KATs have been shown to possess acetyltransferase activity under both in vitro and in vivo conditions [49,50,51]. Interestingly, KAT2A and -2B share a significant degree of sequence similarity but are located on different chromosomes (Table 1 and Supplemental Figure S1). KAT3A and -3B, which also share a greater degree of homology, are master transcriptional coactivators involved in the acetylation of at least two-thirds of the proteome. As shown in Table 1, there are four groups of KATs, (KAT2A/-2B, KAT3A/-3B, MYST, and SRC) [52]. Most KATs mediate acetylation of enhancers in the vicinity of promoters, as well as of intragenic regions of transcriptionally active genes [47]. However, KATs do not act alone; rather, they exist as part of a molecular complex that mediates interactions with the chromatin loop through mediators (Figure 1A,B) [47]. An unbiased proteomics analysis using mass spectrometry revealed that at least 2000 proteins could undergo acetylation, thereby underlining the essential nature of acetylation [49]. Unfortunately, the lack of a prototype epigenetic signature and an overlap in the enzymatic activities of KATs raises several important questions that necessitate the development of novel tools to understand the genome-wide dynamics of acetylation. These questions are centered on elucidating the mechanistic details that will provide insights into the selectivity for protein-protein interactions, which will subsequently determine the biological outcomes.

### 2.1. Cellular Localization of KATs

KATs are conventionally categorized into two distinct groups based on their subcellular localization [53]. Type A KATs (KAT-A) are found in the nucleus and are involved in regulating gene activities via the acetylation of chromatin [54,55,56,57]. Further, KAT-As have a bromodomain (a small modular domain of approximately 110 amino acids) that facilitates the binding of chromatin-associated proteins to the acetylated lysine sites on histone and nonhistone proteins [54,55,56,57]. KAT2A, -2B, -3A, -3B, and -4 are well-known examples of KAT-As that cooperate with activators to enhance transcription.

Type B KATs (KAT-B) are localized in the cytoplasm and are responsible for acetylating the newly synthesized histones before their assembly into nucleosomes [52]. KDACs remove the acetyl groups added to histones by KAT-Bs upon entry into the nucleus and incorporation into chromatin. Interestingly, KAT-Bs lack a bromodomain module [53]. KAT1 is one of the few known examples of a KAT-B involved in histone deposition and chromatin assembly [58]. Despite this discrete classification, KAT proteins function cooperatively as part of multiple complexes. Collectively, mechanisms directing the dichotomy in cellular localization of KATs have not been fully investigated. Table 2 lists the tissues in which KATs are expressed and their involvement in various cancers. While KAT1, -3A, -3B, -4, -5, -6B, -13A, -13B, and -13D are most widely expressed, KAT8 is frequently nondetectable in normal tissues but is overexpressed in lung, breast, and endometrial cancers. These data underscore that investigating the tissue-specific roles of KATs could elucidate new regulatory functions for these enzymes.

### 2.2. Genome-Wide Expression of KATs

**Figure 3:**
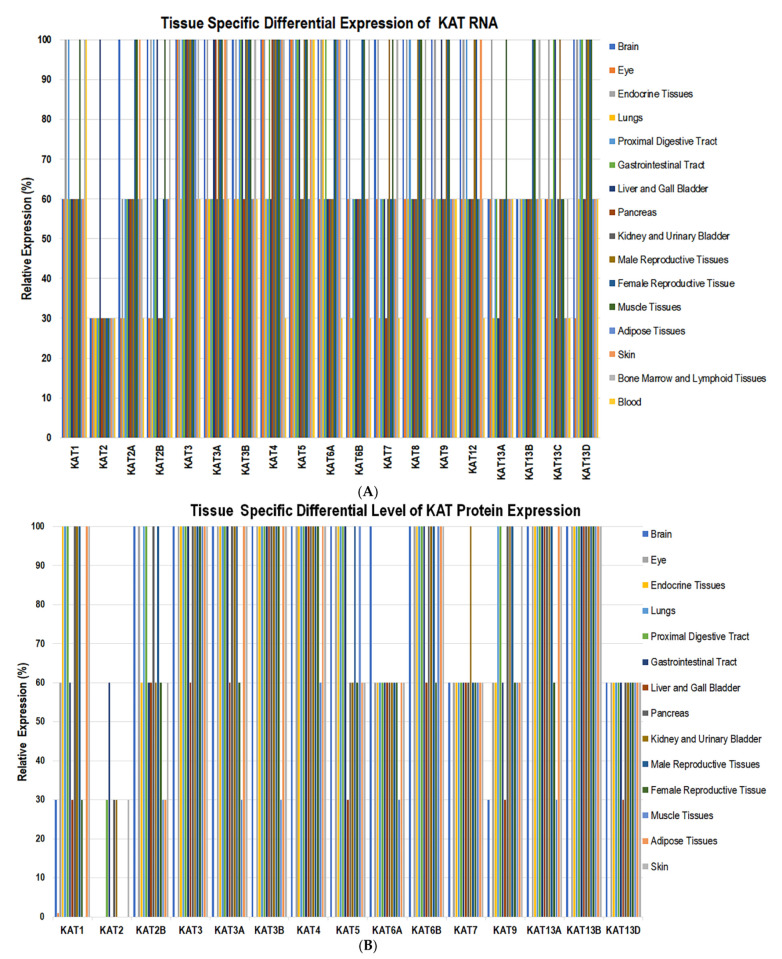
(**A**) Tissue-specific and genome-wide differential expression of KAT RNAs. Low expression corresponds to 30%, moderate expression corresponds to 60%, and high expression corresponds to 100%. (**B**) Tissue-specific and genome-wide differential expression of KAT proteins. Low expression corresponds to 30%, moderate expression corresponds to 60%, and high expression corresponds to 100%.

Engineered mouse models deficient in KAT2A, -3A, -3B, -5, -6A, -6B, or -8 exhibit a lethal phenotype that demonstrates a vital role for the KATs during embryonic development [129,130]. To understand the tissue-wide expression of KATs, websites including human protein atlas [62], uniport [131], antibodypedia [132], metabolic atlas [133] and nextprot [134,135] were reviewed. These websites showcased expression levels for RNA and protein for each KAT. The tissue-specific levels of KATs, RNA, and protein expression were scored as low (30%), medium (60%), and high (100%). The RNA transcripts of *KAT1*, *-2B*, *-5*, and *-8* were found in endocrine tissues, proximal digestive tract, muscle, bone marrow, and lymphoid tissue at high levels. A moderate level of RNA for *KAT1*, *-13A*, and *-13C* was observed in the brain, kidney, and urinary bladder, whereas a lower level of *KAT2* and *-13A* RNA was observed in the lung and gastrointestinal tract (Figure 3A).

Proteins for KAT1, -3, -4, -6B, -13A, and -13B were observed in the lung, proximal digestive tract, kidney, and urinary bladder. The KAT6A, -7, and -13D were expressed in endocrine glands, lung, proximal digestive tract, gastrointestinal tract, kidney, and urinary bladder. KAT1 and KAT9 proteins were expressed in the brain and pancreas (Figure 3B). These findings suggest that the expression levels of KATs themselves are regulated by differential gene expression, cellular and tissue specificity. The RNA levels of *KAT8* are significantly elevated in non-small-cell lung carcinoma and have been linked with the promotion of cell proliferation [102]. KAT5 plays a vital role in generating resistance to chemotherapy in cases of non-small-cell lung carcinoma [81]. The fusion of the *KAT3B* gene and the transcriptional coactivator monocytic leukemia zinc finger protein can cause acute myeloid leukemia [91,92]. These data suggest that alterations in KAT expression negatively affect cellular growth programs.

KAT13A regulates gene expression during puberty—which appears to positively correlate with the overexpression of cellular proto-oncogene tyrosine-protein kinase and cancer progression [127]. KAT13B is found in oocytes and mammary glands [127]. Previous studies have suggested that KAT13B plays a role in both growth hormone regulation and reproductive functions. Additionally, KAT13B has been known to interact with KAT3B and KAT2B. Deletions of KAT13A and -13B have been linked to vascular and skeletal pathologies within these tissues [136].

The KAT13D regulates circadian rhythms by heterodimerizing with the brain and muscle Arnt-like protein-1, a transcription factor that regulates circadian rhythms and relaxes the chromatin via the acetylation of histone 3 [137]. KAT13D deletion has been linked to a decreased lifespan, whereas a *KAT13D* gene polymorphism has been associated with behavioral changes [138]. These behavioral patterns include sleep deprivation, shift work schedule, altered mealtime, and excessive artificial light exposure at night. Interestingly, dysfunction of the molecular clock is linked to uncontrolled cell proliferation in human cancers. Therefore, more knowledge on KAT-directed pathways and strategies to modulate acetylation levels on the chromatin of disease-specific genes will help treat manifestations of developmental syndromes, neurological disorders, and cancers.

### 2.3. Genome-Wide Acetylation Marks on Chromatin

**Figure 4:**
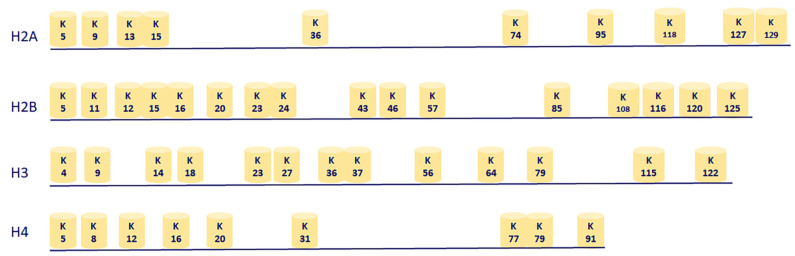
Site- and position-specific acetylation of lysine on nucleosomal histone proteins by KATs serve as a signature for the outcome of transcriptional activity by recruiting either bromodomain or KDACs.

The octameric nucleosome are comprised of ~147 base pairs of DNA wrapped twice around two copies of histone H2A, H2B, H3, and H4 [139]. Lysine acetylation on human histone H2A occurs at positions K5 (H2AK5ac) and K9 (H2AK9ac); on H2B, at positions K5 (H2BK5ac), K12 (H2BK12ac), K15 (H2BK15ac), K16 (H2BK16ac), K20 (H2BK20ac), and K120 (H2BK120ac). On histone H3, acetylation occurs at positions K4 (H3K4ac), K9 (H3K9ac), K14 (H3K14ac), K18 (H3K18ac), K23 (H3K23ac), K27 (H3K27ac), K36 (H3K36ac), and K56 (H3K56ac). On histone H4, acetylation occurs at positions K5 (H4K5ac), K8 (H4K8ac), K12 (H4K12ac), K16 (H4K16ac), K20 (H4K20ac), and K91 (H4K91ac) (Figure 4) [6,20,39,47].

The presence of H3K9ac, H3K56ac, and H2AK9ac on promoters serves as marks for transcriptionally active genes [6,20,39,47]. Similarly, transcriptionally active enhancers are enriched with the H3K27ac mark. The presence of KATs in the vicinity of an active gene is not restricted to a simple enzymatic function, but it is instrumental in promoting complex molecular assembly. Inhibitors of KDACs activate transcription, indicating that the coregulatory activities of KATs and KDACs on the chromatin modulate promoter activities. A recent report suggested that H4K20ac was associated with low expression levels on promoters that do not overlap with transcriptionally active genes [6,20,39,47]. However, the coactivators KAT3A and KAT2B are recruited to H4K20ac via their acetyl-lysine-binding bromodomains. Furthermore, the H4K20 site underwent trimethylation by Pr-SET, which binds strongly to the chromodomain, in a *Drosophila* model [6,20,39,47]. H4K20me3 mediated by SUV4-20H2 in humans is involved in epithelial-mesenchymal states in pancreatic cancer [140]. Taken together, while it is possible to pull down large protein complexes, determining the role of individual domains and the timing of acetylation and deacetylation requires integration of combinatorial approaches, including small molecules, clustered regularly interspaced short palindromic repeats (CRISPR) gene-editing systems, and real-time ChIP assays.

### 2.4. Sequence Analysis of Acetyltransferase Domain of KATs

**Figure 5:**
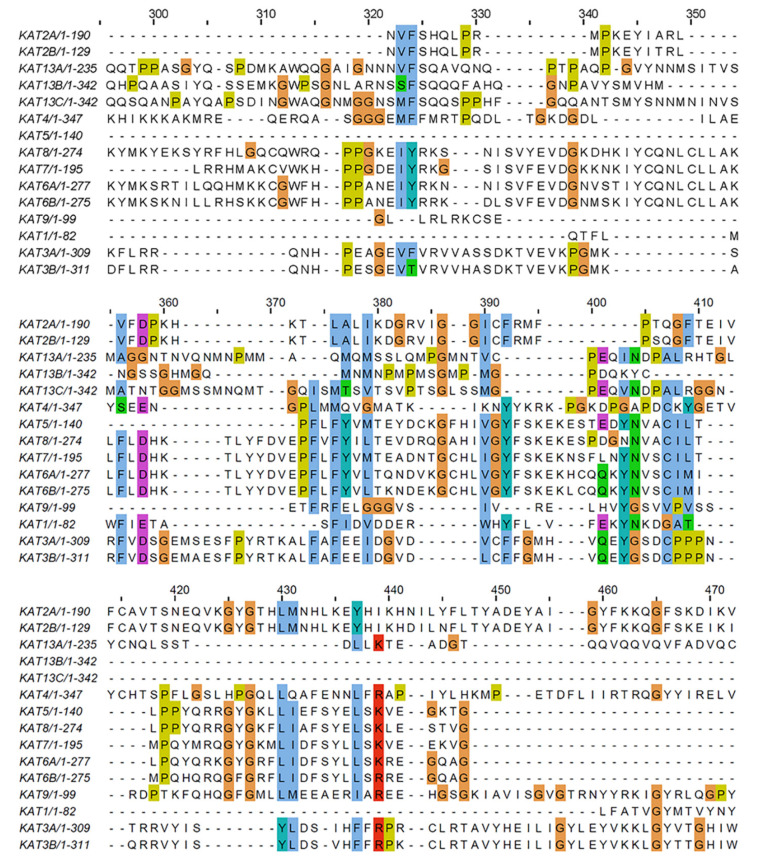
Partial multiple sequence alignment of the acetyltransferase sequences from 15 KATs that show the most sequence similarity/identity. The sequence numbers refer to the full alignment.

The overall sequence similarity amongst KATs was low. While the longest KAT sequence had 347 residues, the multiple sequence alignment extended to 650 residues. This extension was mainly because the KAT13 and KAT3A/-3B families aligned rather well, but they did not align with the MYST (KAT5, -6A, -6B, -7, and -8) and Gcn5-related N acetyltransferases (GNAT) (KAT2A and -2B) families. Supplementary Figure S1 shows the full multiple sequence alignment, and Figure 5 shows partial alignment with the highest degree of sequence similarity.

**Figure 6:**
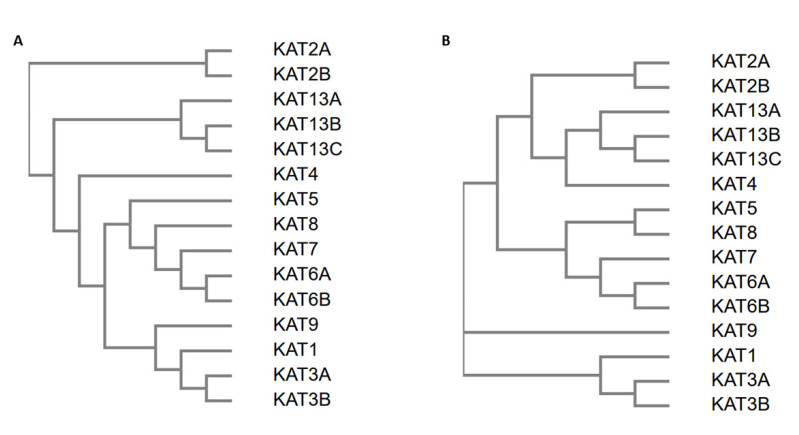
Hierarchic clustering of the KAT sequences. Panel (**A**) represents the guide tree, and panel (**B**) represents the phylogenetic tree.

The European Molecular Biology Laboratory (EMBL) analysis also generated a guide tree (i.e., hierarchical clustering based on the pairwise alignment scores) as well as a phylogenetic tree (i.e., hierarchical clustering based on the multiple sequence alignment scores), as shown in Figure 6A,B, respectively [141,142,143]. This figure also shows the family to which the sequences belong (GNAT, MYST, steroid receptor coactivator (SRC), or CREB-binding protein (CBP)/p300). The families SRC, MYST, and CBP/p300 were clearly separated into different branches of both trees. However, the GNAT family had members in two branches; KAT1 (the only GNAT family member not associated with bromodomains) was in the CBP/p300 family. Comparing the two trees, the phylogenetic tree produced more subclusters than the guide tree, thus providing additional information about these protein domains (Figure 6A,B).

### 2.5. Structural Analysis of Acetyltransferase Domain of KATs

**Figure 7:**
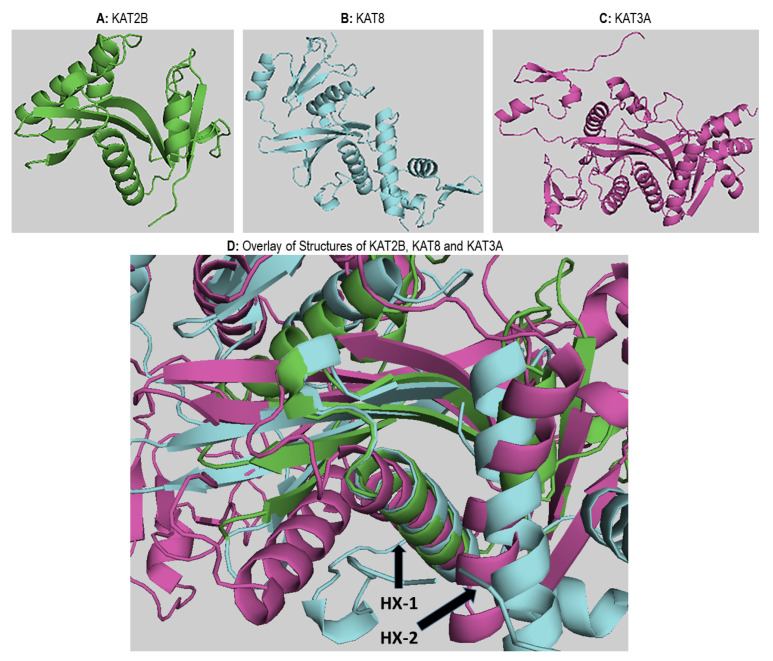
Cartoon representation of the KATs representative of the class. Panel (**A**) represents KAT2A. Panel (**B**) represents KAT8, and panel (C) represents KAT3A. Panel (D) represents the superimposition of KAT2A, KAT8, and KAT3A family representatives, showing the binding site region.

To compare the structures of the different KAT families, representative structures were aligned. The crystal structures were obtained from the Protein Data Bank (PDB) [144,145,146], which involved a multiple sequence alignment to the smallest acetyltransferase domain (KAT2A) [147]. This alignment of the corresponding atoms was needed for the overlay. Figure 7A–C show the structures of KAT2A (PDB id: 1cm0), KAT8 (PDB id: 2giv), and KAT3A (PDB id: 5u7g), respectively. The binding site is shown in Figure 7D with the three structures superimposed. In all panels, the binding site is facing the viewer. The KAT-binding site is bordered by a beta-sheet composed of three strands and by two alpha helices (HX1 and HX2). In KAT2A, one of the beta strands was formed by residues 51–86, the longer alpha helix (HX1) is formed by residues 92–109, and the shorter alpha helix (HX2) is formed by residues 121–130. These helices are labeled in Figure 7D as HX1 and HX2, respectively. Interestingly, the structural alignment was nearly perfect for the beta-sheet—especially for HX1—but significantly worse for HX2. This observation was also consistently noted when several family members were used for the structural alignment (data not shown). In fact, the sequence alignment did not match any of the aligned residues to the shorter helix of KAT2A. However, when other KAT3A structures were added to the alignment (data not shown), the helices that matched the shorter helix of KAT2A aligned very well within the family. These findings suggest that the shorter helix is responsible for the specificity of each KAT.

### 2.6. Mutually Exclusive Crosstalk between Epigenetic Marks

**Figure 8:**
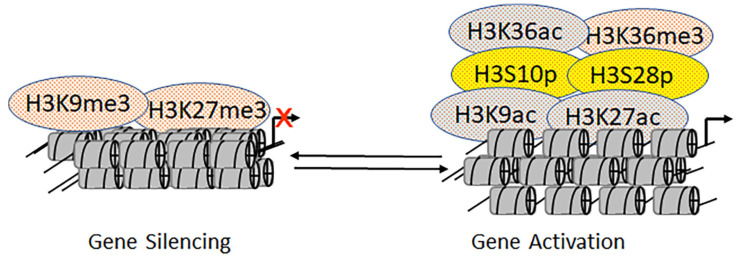
Mutually exclusive acetylation and methylation on lysine residues H3K9 and H3K27. Cross-talks between H3K9 and phosphorylation at H3S10 and between H3K27 and phosphorylation at H3S28. Together, these epigenetic modifications unfold mechanisms, which are responsible for cellular response to extracellular signals.

Mutually exclusive epigenetic modifications on the histone proteins of the enhancer and promoter regions facilitate activation or repression [5]. For instance, H3K9 or H3K27 sites could undergo either trimethylation (me3) or acetylation. While H3K9me3 and H3K27me3 are hallmarks of gene repression, H3K9ac and H3K27ac indicate gene activation (Figure 8) [148]. These mutually exclusive events play a crucial role during development and embryogenesis in multicellular organisms [149,150]. Additionally, H3K9 and H3K27 can undergo biotinylation and sumoylation [151]. However, the functional readouts of these modifications are not yet fully understood. Similarly, the biotinylation of H4K12 blocks the activation potential of H4K12ac [152]. Although mutually exclusive modifications on histone tails induce antagonistic effects, such is not the case with H3K36: Both H3K36ac and H3K36me3 activate downstream transcriptional events [14,153]. In transcription factors like p53, acetylation is mutually exclusive to methylation and ubiquitination on lysine 382 [154,155,156]. We still do not know whether there are separate pools of differentially modified p53 and histone proteins in cells which different—or the same. The ability of lysine residues to undergo mutually exclusive modifications makes the acetylation process more complex than kinase-mediated phosphorylation.

Phosphorylation is integral to the site-specific and combinatorial epigenetic modifications on the nucleosomal histone proteins [157]. The phosphorylation of histone H2A(X) flags the nucleosomes near the site of DNA damage [157]. Moreover, phosphorylation at serine 10 on histone H3 (H3S10p) and serine 32 on histone H2B are associated with epidermal growth factor-responsive gene transcription. H3S10p and phosphorylation of serine 28 on the histone H3 (H3S28p) play a pivotal in controlling the expression of proto-oncogenes [157]. In *Tetrahymena*, the biological role of H3S10p is associated with mitosis, and the level of H3S28p is induced by ultraviolet B treatment. Previous studies have defined the positive effects of histone H3 phosphorylation on the growth factor-mediated expression of the *c-fos* and *c-jun* proto-oncogenes [158]. Subsequently, the three-dimensional, ternary complex structure of the *Tetrahymena* KAT2A domain—bound to both acetyl-CoA and H3 peptide—revealed major interactions between its catalytic site and the H3 peptide [40,41,159,160,161]. Later, biochemical analysis of this structure revealed that the phosphorylated histone H3 had a higher binding affinity for KAT2A than the unphosphorylated peptide. Further, it was established that the H3S10p facilitated the H3K9ac [40,41,159,160,161,162]. However, it is not clear whether H3S10p and H3K9ac coexist on the same histone tail. Furthermore, using a dual-specific antibody that unambiguously recognizes both H3S10p and H3K9ac, H3S10p was confirmed to be essential for the acetylation of H3K9. Interestingly, the SUV39h1-mediated methylation of H3K9 inhibited H3S10p. Finally, in a yeast model system, the NuA4-mediated acetylation occurring at lysine 5, 8, 12, and 16 on histone H4 was inhibited by phosphorylation on serine 1 by Casein kinase 2 which is believed to exist in a complex with KDACs to enhance deacetylation [163].

In addition to the crosstalk between phosphorylation and lysine acetylation, there is cooperativity between acetylation and arginine methylation. During the transcriptional activation of nuclear receptor p160, the coactivator-arginine methyltransferase 1 interacts with KAT3A, which leads to the methylation of arginine 17 and the acetylation of lysine residues 18 and 23 on histone H3 [162]. We understand the significance of crosstalk between neighboring modifications better than distant modifications from antibody studies. Together, data from mass spectrometry and antibody-based investigations may not fully reveal the existence of different pools of nucleosomes undergoing differential modifications. The use of highly selective small molecules and specific antibodies with real-time live imaging technologies could prove to be useful to address these questions.

## 3. Acetylation of Nonhistone Proteins

### 3.1. Metabolism, Developmental Disorders, and Cancers

**Figure 9:**
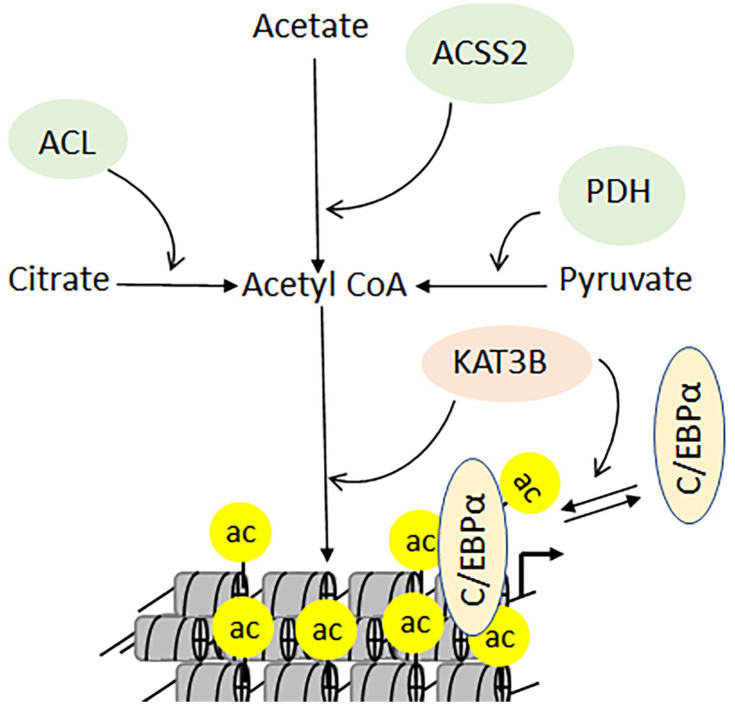
The acetyl-CoA is a key metabolite that could be generated by three different enzymes. Pyruvate dehydrogenase complex (PDH) generates acetyl-CoA in mitochondria, whereas ATP-citrate lyase (ACLY) and acyl-CoA synthetase short-chain family member 2 (ACSS2) generate a nonmitochondrial pool of acetyl-CoA.

High-resolution mass spectrometry and bioinformatics analyses have revealed that acetylated proteins are detectable in the nucleus and cytoplasm, as well as in the endoplasmic reticulum and mitochondria [49,50,164]. The presence of acetylated proteins in mitochondria raised questions about the molecular link between acetylation and metabolism, which has been recently established [49,50,164]. For example, one of the first indications of such a relationship emerged from the role of acetyl-CoA as a cofactor in lysine acetylation—and as a metabolite bridging glycolysis with the tricarboxylic acid cycle. Biochemical data confirmed that the depletion of acetyl-CoA reduces the levels of cellular acetylation [165]. The enzyme acetyl-CoA synthetase short-chain 2 (ACSS2), which converts acetate into acetyl-CoA, interacts with KAT3B, resulting in enhanced chromatin acetylation [166]. This interaction is indispensable for the differentiation of neuronal cells, as the knockdown of ACSS2 causes reduced acetylation and loss of memory in mice [164]. Furthermore, in the presence of high glucose levels, the transcription factor C/EBPα is acetylated by KAT3A, which induces its transcription function [167]. Conversely, the deacetylation of C/EBPα stimulates the expression of genes involved in mitochondrial biogenesis, stimulating ATP production to maintain energy levels under low-glucose conditions. In addition to ACSS2, pyruvate and citrate are converted by the pyruvate dehydrogenase complex and ATP-citrate lyase into acetyl-CoA (Figure 9).

Congenital mutations in KATs can cause developmental disorders, intellectual challenges, cardiac problems, and craniofacial, genital, and behavioral abnormalities [47]. Notably, these mutations do not impact the acetyltransferase function of KATs. Still, they do influence protein-protein interactions and the formation of molecular complexes that work in tandem with the general transcriptional machinery [47]. These mutations are present in KAT3A, -3B, -6A, and -6B. However, mutations in KAT3A lead to Rubinstein–Taybi syndrome, impacting the level of acetylation [47]. The dysfunctional KAT complexes tilt the stoichiometric balance towards KDACs. Therefore, calibrating the use of KDAC-dependent inhibitors could elicit a potential therapeutic advantage in certain clinical situations.

**Figure 10:**
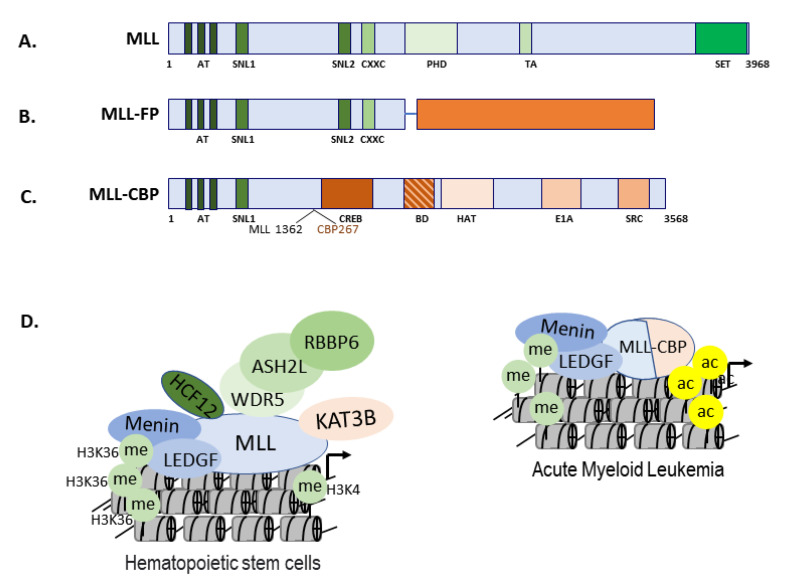
N-terminal of *MLL* fuses with the bromodomain and histone acetyltransferase domain (HAT) domain of *CBP/KAT3A* to form MLL-KAT3A fusion protein that has the potential to cause acute myeloid leukemia (AML). Panel (**A**) represents the full-length multidomain MLL protein. Panel (**B**) represents a typical MLL fusion protein (MLL-FP) formed mainly by the N-terminal domain of MLL. Panels (**C**,**D**) represent the constitution of MLL-KAT3A fusion protein and highlight the altered chromatin landscape that changes the cell fate of hematopoietic stem cells towards AML.

The acetylation-mediated function of KATs (supporting either tumor suppression or uncontrolled growth) is dependent upon the upstream signals, cell types, and types of mutations involved [168]. Somatic mutations in KATs that deregulate the level of acetylation also result in oncogenesis [169]. Genetic translocation leading to the expression of fusion proteins is a major mechanism by which cells gain uncontrolled self-renewal and growth properties [170,171]. The myeloid/lymphoid or mixed-lineage leukemia protein (MLL) regulates gene expression during early development and hematopoiesis by trimethylation at lysine 4 on histone H3 (Figure 10A) [172]. In murine models, deregulated MLL causes anxiety and cognitive defects [173]. Additionally, MLL demonstrates a higher tendency toward chromosomal translocation that generates different types of MLL fusion proteins (MLL-FP; Figure 10B). The fusion of MLL with KAT3A causes acute lymphoblastic leukemia and acute myeloid leukemia, respectively (Figure 10C) [170,174,175]. In normal situations, MLL and KAT3A cooperate during the differentiation of hematopoietic stem cells that are deregulated upon the formation of MLL-KAT3A fusion protein [170,174,175]. The MLL-KAT3A can cause acute myeloid leukemia (Figure 10D) [170,174,175]. Missense, inactivating, and truncation mutations within *KAT3A/-3B* may cause breast, gastric, ovarian, and small-cell lung cancers as well as diffuse, large cell, and follicular lymphoma [72]. Deletion, amplification, and translocation of the *KAT6B* gene can lead to leiomyoma and bladder, colorectal, and small-cell lung cancers [176]. Together, these data suggest that the functions of KATs have been characterized better in disease situations than in normal cells.

### 3.2. Target Acetylation Pathway in Nuclear Factor-Kappa B-directed Chronic Diseases

**Figure 11:**
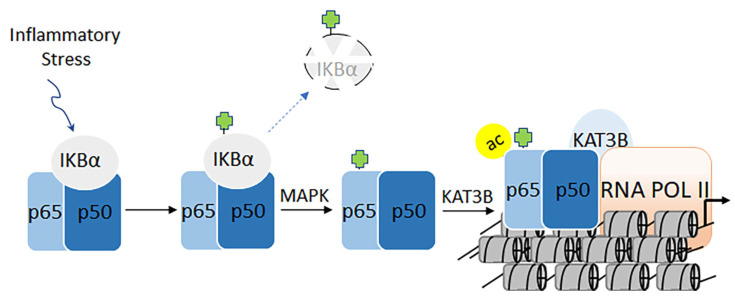
Proinflammatory signals trigger phosphorylation followed by ubiquitination-mediated degradation of IκBα. This dissociation of the ternary complex releases p65/p50 from IκBα heterodimer. Subsequently, the phosphorylation of p65 on serine 276 (yellow balls) by the mitogen-activated protein kinase leads to acetylation of lysine 310 on p65 (green balls), which potentiates the transcriptional activity of NF-κB.

The nuclear factor kappa-light-chain-enhancer of activated B cells (NF-κB) family of master transcription factors plays a central role in expressing genes that regulate innate and adaptive immune responses [177,178,179]. The induction of NF-κB is triggered by inflammatory signals, bacterial and viral infections, free radicals, heavy metals, and ultraviolet light [177,178,179]. The dysregulation of NF-κB transcriptional activities leads to cancers, acute and chronic inflammation, autoimmune disorders, and septic shock [180]. Classical activation of NF-κB involves the release of the cytoplasmic p65/p50 heterodimer from IκB, which facilitates its nuclear localization and interaction with coactivators on the promoters of immune-response genes, leading to a cellular response (Figure 11) [181]. While the phosphorylation of IκB leads to its ubiquitination, the phosphorylation of p65 leads to its nuclear localization [182]. The interaction of p65/p50 with coactivators plays a crucial role in modulating downstream genes that mediate appropriate cellular responses depending on the type and level of cellular stress [183]. The molecular interplay between KAT3A/-3B and Rel A epitomizes a critical aspect of NF-κB biology: KAT3A/-3B has been shown to acetylate Rel A on lysine 310 (RelAK310ac) [182]. Together with its phosphorylation on serine 276, RelAK310ac plays a major role in recruiting the positive transcriptional elongation factor P-TEFB. Conversely, the methylation of lysine 310 by SETD6 proteins facilitates the interaction of Rel A with the histone methyltransferase GLP/G9a, leading to the downregulation of transcription (Figure 11) [184,185]. Recently, several reports have established a pivotal link between acetylation and the activation of NF-κB-directed pathways, which are central to the development of cancers and chronic inflammatory bowel disease. One key protein that has emerged to modulate the transcriptional activities of NF-κB is BRD4, which is a member of the bromodomain and extra-terminal domain families [186]. The tandem double bromodomain with BRD4 has the capacity to bind acetylated chromatin as well as NF-κB [187]. BRD4 has been shown to regulate the levels of enhancer RNA [188]. Given these multiple roles, BRD4 has emerged as a potential drug target. Reports have also suggested that BRD4 inhibitors may suppress the activation of TNFα, IL-6, IL-17A, and IL-8 [189,190].

### 3.3. Multifaceted Roles of p53 C-Terminal Domain during Genotoxic Stress

**Figure 12:**
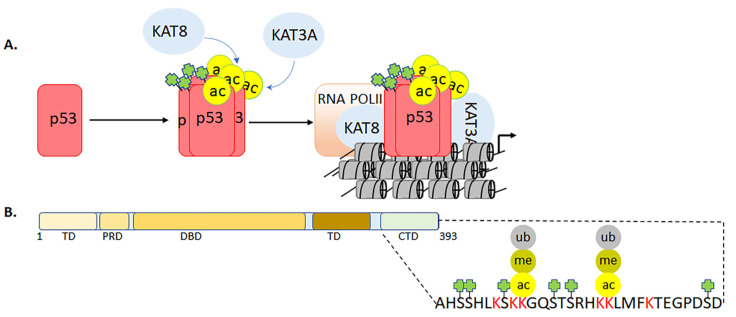
KAT3A and 3B regulates the tumor suppressor functions of p53 by molecular interaction and acetylation on the C-terminal doman (CTD). Panel (**A**) represents the activation of p53 under genotoxic stress which results in the phosphorylation at serine 15 (green box) and acetylation of p53, at lysine 120 by KAT8 and lysine 382 by KAT3A/3B (yellow circles). These modifications are critical for the stability and transcriptional activation of p53. Panel (**B**) represents the C-terminal tails of p53 that—like histone tails—serve as modification and recruitment sites for coactivators.

The role of tumor suppressor protein p53 has ranged from a viral antigen to a central player in regulating cellular responses to a wide range of genotoxic stresses over the past four decades [191,192,193]. Recent investigations have systematically established that the functions of p53 extend beyond the regulation of apoptosis and cell cycle arrest to include biological processes such as metabolism, oxidative balance, aging, autophagy, and ferroptosis [154,156]. Epigenetic modifications and protein-protein interactions are the major determinants that confer multitier gene regulatory capabilities to p53 [194]. The tumor suppressor protein p53 was also discovered to be one of the first nonhistone proteins that could undergo KAT-mediated acetylation on its C-terminal domain (CTD) [195,196]. The other major epigenetic modifications of p53 include phosphorylation, ubiquitination, and methylation [154,156]. Additionally, sumoylation, neddylation, O-GlcNAcylation, adenosine diphosphate ribosylation, hydroxylation, and β-hydroxybutyrylation have been shown to modulate the transcriptional activities of p53 [154,156]. During genotoxic stress, activation of p53 involves its phosphorylation on serine 15, which rescues p53 from the Mouse double minute 2 homolog (MDM2)—a major E3 ubiquitin ligase and negative regulator of p53 (Figure 12A). Subsequently, the lysine 382 on the p53 CTD undergoes acetylation by KAT3A/-3B (p53Kac382). Notably, MDM2 can ubiquitinate p53 at six lysine residues within the CTD (K370, K372, K373, K381, K382, and K386; Figure 12B). High levels of MDM2 activity promote p53′s polyubiquitination and nuclear degradation, whereas low levels induce monoubiquitination and nuclear export. However, in the cytoplasm, p53 can perform transcription-independent roles [154].

Three unstructured regions within the p53 protein are located within the N-terminal domain between the DNA-binding and oligomerization domains and CTD [55,194,197]. Furthermore, the unstructured CTD serves as a major recruitment platform for coactivators—and has been shown to exhibit various secondary conformations during these molecular interactions. Additionally, the CTD has been shown to bind DNA independent of any sequence specificity. The major sites of acetylation on the CTD are K320, K372, K373, K381, and K382 (Figure 12B) [55,194,197]. The K382, which can be acetylated by KAT3A/-3B, has gained increased attention, possibly because of the availability of robust antibodies that recognize this site. The p53K382ac serves as a docking site for the bromodomain-mediated recruitment of KAT3A/-3B and the induction of the downstream target CDKN1A leads to growth arrest [55,194,197]. In addition to its interaction with bromodomains, the acetylation of p53 has been shown to impact DNA binding and cellular localization [198]. Furthermore, acetylation at K120 by KAT8 plays a crucial role in p53-mediated apoptosis [155,199]. The synergy between acetylation at K164 by KAT3A/-3B and K120 acetylation contributes to p53-directed cell cycle arrest [153,199]. More recently, the p53-3KR mouse model that expresses acetylation-deficient p53 (K117/161/162R)—which mirrors K120/164R mutations in human p53—was used to demonstrate that while the apoptotic and growth arrest functions of p53 were lost, p53-dependent metabolic regulation was still intact [191]. Unlike wild-type p53, the CTD-deleted p53 lacks the ability to bind DNA and activate downstream target genes (Figure 12B). Collectively, biochemical, cellular, and animal models have provided major answers regarding the biological role of p53. However, the underlying mechanisms that direct crosstalk between acetylation and various modifications of p53 for activating downstream target genes selectively require further investigation. One possibility would be to take advantage of the CRISPR gene editing system to mutate the active site of the KATs and use small molecules against the conserved modular domains of p53 coactivators.

### 3.4. The Role of MYST Family in Gene Regulation and Cellular Response

**Figure 13:**
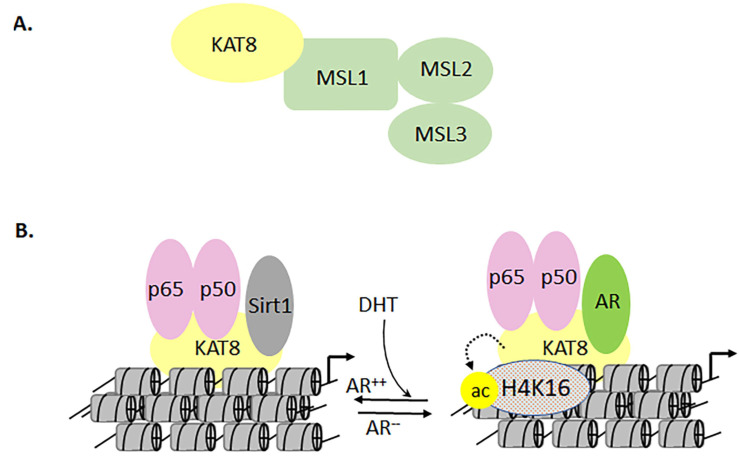
Differential molecular interactions mediated by KAT8 lead to modulation of downstream target genes. Panel (**A**) represents the interaction of KAT8 with MSL1, MSL2, and MSL3. Panel (**B**) represents the dichotomy in MYST interactions with NF-κB and AR in prostate cancer cells.

The MYST family of KATs (which includes KAT5, -6A, -6B, -7, and -8) plays a central role in maintaining cellular homeostasis and stem cell patterning during embryogenesis [102,129,176]. These KATs are also involved in regulating DNA damage responses, cell cycle control, and apoptotic pathways [47]. The genome-wide screening of KATs revealed that KAT6A is most susceptible to having varying copy numbers, enabling cells to exhibit a tumorigenic phenotype [200]. A KAT6A-TIF2 fusion protein suppresses the expression of *CDKN2A*, which leads to the downregulation of cellular senescence [93]. The human KAT6A gene was first identified in patients with acute myeloid leukemia [176]. The KAT6A and -6B genes are composed of plant homeodomain, serine-rich, and methionine-rich domains. In the studied patients, KAT6A formed in-frame fusion proteins with KAT3A or -3B. Further, the KAT6A-TIF2 fusion was shown to provide hemopoietic stem cells with the sustained potential for self-renewal during leukemic transformation [93]. Experimental models have revealed that while KAT6A is important for the differentiation of hematopoietic stem cells, KAT6B is essential for the differentiation of neuronal cells into neurons, astrocytes, and oligodendrocytes [201]. KAT5 was one of the first members of the MYST family to be identified for its ability to interact with the HIV transactivator protein (Tat) [202]. Interestingly, interactions between KAT5 and HIV Tat prevent apoptosis, thereby indirectly supporting HIV persistence in immune cells [202]. Among the MYST family, KAT5 is most closely related to KAT8 which, when mutated, affects only male flies. Breast, ovarian, colorectal, renal, gastric, and hepatocellular cancers exhibit lower levels of KAT8 protein and H4K16ac than their normal tissue counterparts [104,203,204,205]. These findings are associated with poor prognosis and increased risk of metastasis in these tissues. Besides, male-specific lethal proteins 1, 2, and 3 (MSL1, MSL2, and MSL3) form a complex with KAT8 to mediate H4K16ac [47]. The biological significance of this complex was first investigated in a *Drosophila* model of dosage compensation. Mutations in MSL3 affect the level of H4K16ac [47]. However, compared to KAT8, the specific roles for MSL1, MSL2, and MSL3 are still not well understood. In addition to the acetylation of H4K16, H4K20 can undergo mono-, di-, or trimethylation—or acetylation. However, the crosstalk between these neighboring modifications is not clear. KAT8 has the ability to be a part of two different complexes: the MSL-1 and the MSL1v1 complexes [11]. Furthermore, the role of the methyl-lysine-binding chromodomain has yet to be elucidated in translational models (Figure 13A).

In PCa, the functional synergy between the AR and NF-κB has the potential to increase the chemoresistance to antiandrogens that could promote aggressive tumor growth [33,206,207]. Although the underlying mechanisms are less clear, the regulatory abilities of coactivators can bridge the transcription functions of both AR and NF-κB. Indeed, KAT8 has been shown to costimulate AR and NF-κB functions in PCa cells [33]. A report also demonstrated that the activation of NF-κB promoted the deacetylation of KAT8 by NAD-Sirtuin 1. Furthermore, the mutually exclusive interactions between KAT8 and NAD-Sirtuin 1 or AR regulate the acetylation of lysine 16 in histone H4 [33]. Notably, in AR-negative PC3 cells (as well as in AR-depleted LNCaP cells), the downregulation of KAT8 activates the cleavage of poly ADP ribose polymerase (PARP) and caspase 3, leading to apoptosis. In contrast, in AR-expressing PC3 cells (PC3-AR), the depletion of KAT8 induces CDKN1A/p21 expression, which results in G2M arrest. Concomitantly, the levels of phosphorylated retinoblastoma, E2F1, CDK4, and CDK6 proteins are all reduced. Additionally, the expression of tumor protein D52 is unequivocally affected in PC3, PC3-AR, and LNCaP cells, suggesting that the functional interactions of KAT8 with AR and NF-κB are critical for PCa progression (Figure 13A,B) [33].

### 3.5. KAT2A and -2B in Transcriptional Regulation of HIV Activation

**Figure 14:**
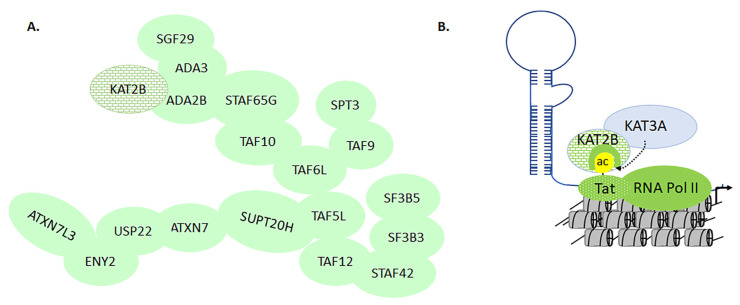
Differential molecular interactions mediated by KAT2A/-2B lead to modulation of downstream target genes. Panel (**A**) represents the interaction of KAT2A/-2B with 700 kDa general transcriptional factors. Panel (**B**) represents the dichotomy in KAT2B interactions with HIV Tat acetylated at lysine 50. Acetylation of Tat by KAT3B facilitates bromodomain-mediated recruitment of KAT2B.

In human cells, KAT2B forms a complex with Ada2, Ada3, Spt3, PAF400, PAF65β, TAF15/20, PAF65α, TAF30, and TAF31 [47]. Although the acetylation activities of KAT2A and -2B were characterized early on, the epigenetic signature of these complexes remains to be determined (Figure 14A) [47,208]. HIV Tat is a 102-amino acid polypeptide that plays a central role in the transcriptional activation of HIV, which occurs on the long terminal repeat of the proviral DNA. Tat initiates transcription by interacting with the transactivation response element (TAR). KAT2A acetylates four lysine residues at the C-terminal of HIV integrase, including K258, K264, K266, and K273 [209]. Knocking down KAT2A reduces HIV integration, leading to a decline in infectivity [209]. Additionally, K264, K266, and K273 are acetylated by KAT3B. KAT2B regulates transcriptional activation by acetylation of lysine 28 on HIV Tat (TatKac28) and binds to Tat acetylated lysine 50 (TatKac50) through its bromodomain. While TatKac28 helps the recruitment of Tat to the CDK9/PTEF-B complex, the TatKac50 helps to dissociate Tat from TAR, which leads to enhanced HIV Replication [54]. The bromodomain of KAT2B competes for the TatKac50 site, which helps Tat/TAR dissociation. Together, these epigenetic changes promote chromatin remodeling that further accelerates the replication and expression of HIV proteins that devastate the human immune system (Figure 14B) [54,210]. Finally, blocking the acetyltransferase module of KAT3A/-3B as well as the KAT2B bromodomain with small molecules reduces Tat-mediated activation of HIV replication [211].

### 3.6. The Dynamics of KATs and KDACs in Models of Traumatic Brain Injury

**Figure 15:**
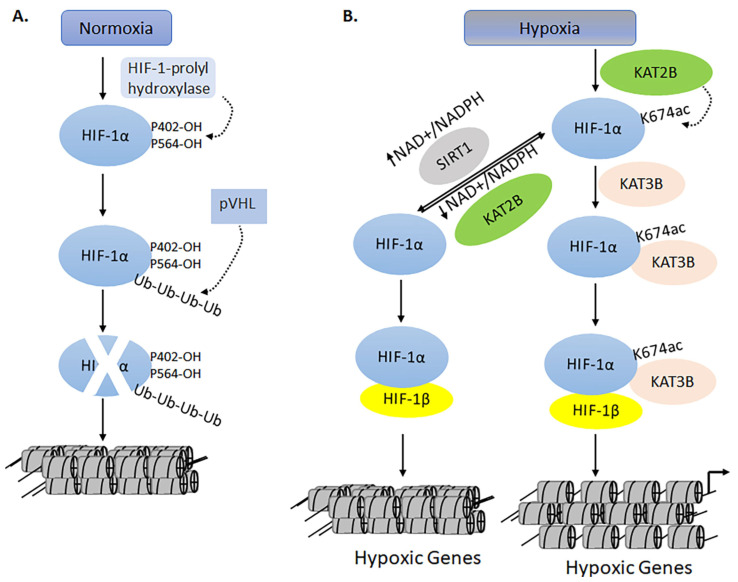
Molecular interplay of acetylation, deacetylation, and ubiquitination by KATs, KDAC, and ubiquitinases on the oxygen-sensitive transcription factor hypoxia-inducible factor (HIF)-1α. Panel (**A**) represents the ubiquitination-mediated degradation of HIF-1α when oxygen is normal. Panel (**B**) represents the acetylation-mediated stabilization and transcriptional activation of HIF-1α.

The brain is one of the most metabolically active organs and is critically dependent upon normal physiological levels of oxygen. Traumatic brain injury (TBI) from sudden accidents or cardiac arrest can cause immediate oxidative stress, acute inflammation, apoptosis, the indiscriminate release of neurotransmitters, and unchecked ionic influxes [212,213]. These cellular imbalances result in severe cerebral hypoxia, an acute medical condition that reduces the oxygen circulation to the brain. It is often fatal. Administration of valproate to patients with TBI (inhibiting KDACs that would lead to increased acetylation) underlines the crucial interplay between KATs and KDACs [214]. Mice models suggest that histone acetylation is causally related to memory enhancement [215]. An earlier report suggested that histone acetylation may reverse ischemic brain injury in neonatal rats [27]. Another report by Gao et al. demonstrated that TBI reduced the levels of histone H3 acetylation in the hippocampal region [216]. Taken together, these epigenetic changes affect the hypoxia-regulated genes. Hypoxia-inducible factors (HIFs) are the major transcription factors that respond to declines in cellular and physiological oxygen levels by regulating gene expression during hypoxia [27]. HIFs are hydroxylated on proline residues under normal oxygen conditions and are later degraded by an oxygen-dependent proteasomal pathway mediated by the von Hippel–Lindau protein (Figure 15A) [217]. However, under hypoxic conditions, KAT2B acetylates HIF-1α on lysine 674, which activates hypoxia-responsive genes in the presence of KAT3B (Figure 15A,B) [217]. Transcriptional regulation by KAT3A and -3B enhances acetylation-mediated hypoxic gene expression [216]. HIF-1 is a heterodimer that consists of alpha and beta subunits [218]. The upregulation of HIF-1α has been shown to improve tolerance to hypoxia and reduce neuronal cell loss. The NAD-dependent NAD-dependent Sirtuin 1 deacetylates HIF-1α, which loses its ability to interact with KAT3B to activate hypoxia-regulated genes. Additionally, NAD--dependent Sirtuin 1 plays a significant role in the maintenance of mitochondrial function in the brains of zebrafish during hypoxia. The NAD--dependent Sirtuin 1 increases oxidative metabolism due to the inhibitory effects of acetylation by reversing deacetylation (Figure 15A,B) [218]. Clearly, we are only beginning to probe the complex relationships between KATs and KDACs in normal brain/neurological disorders. Although gene knockout models will provide a broad look at the functional significance of KATs and KDACs, the use of CRISPR and small molecules will give a better understanding of brain function during normal and disease-affected situations.

In summary, KATs orchestrate acetylation-dependent and acetylation-independent mechanisms that are multilayered and intertwined with the functions of transcription factors activated by upstream cues. Additionally, the biological role of integrates gene regulation, metabolic processes, and stress-directed cellular responses. Given their essential role in human biology, a combinatorial approach is the key to designing tools for unraveling the complete functions of KATs. One approach could be to develop highly selective small molecules to target KATs utilizing computational and chemical biology approaches.

## 4. Chemical Biology of Acetylation-Mediated Mechanisms Directed by KAT3A/-3B

Advancements in sequencing technology and data from the human genome project have reached a zenith in rapidly detecting mutations and diagnosing human genetic diseases. However, understanding the role of the epigenetic basis of disease pathogenesis is challenging, due to the involvement of an array of posttranslational modifications and molecular interactions. Most chromatin-associated proteins, including KATs, consist of multiple domains that interact with the transcriptional machinery in more than one fashion. Determining the influence of the KAT-associated protein complexes on their enzymatic activity and substrate specificity is challenging. Additionally, the enzyme kinetics of a recombinant KAT may not be consistent with their in vivo activity. This may limit data interpretation from in vitro assays with in vivo disease models. Together, gene manipulation may not fully reveal a modular domain’s role in mediating epigenetic modifications versus molecular interactions. A small molecule can block the specific function of a target protein without affecting its endogenous expression, unlike microRNA, small interfering RNAs and gene knockout-based approaches [219,220]. Furthermore, small molecules have an added advantage, in that they can easily enter the cells without transfection or electroporation because of their low molecular weights [221]. Small molecules targeting bromodomains have been shown to negatively affect the level of acetylation [56,194,222,223]. Such investigations increase our knowledge regarding the mechanisms involved in the assembly of activator-versus-repressor complexes on the gene promoter.

KAT3A/-3B-mediated H3K27ac serves as a marker for gene activation [224]. However, H3K27me3 by EZH2 leads to gene silencing [225]. Thus, KAT3A modulators can control gene activation versus silencing on disease-specific promoters. A study using human melanocytes showed that downregulation of KAT3A activity inhibited growth and induced cellular senescence [226]. Additionally, depending upon the cellular context, KAT3A activity is required for the G1/S transition of the cell cycle [73]. KAT3A/-3B undergoes chromosomal translocation, causing myeloid leukemia [227]. KAT3A also serves as a pharmacological target for minimizing p53-induced pathology in normal tissues. Below, we discuss the potential of inhibiting the acetylation-directed functions of KAT3A/-3B by small molecules A485, C646, and nitrile-curcuminoid (NiCur) in various model systems.

### 4.1. A485

**Figure 16:**
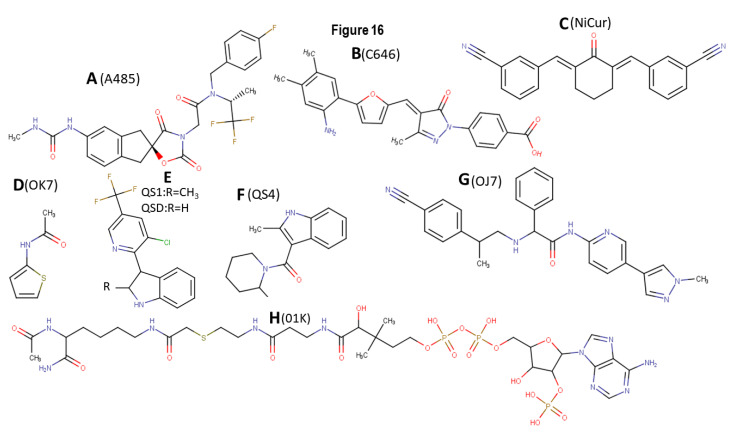
Chemical structure of the ligands: (**A**) A485, (**B**) C646, (**C**) NiCur, (**D**) 0K7, (**E**) QS1 and QSD, (**F**) QS4, (**G**) OJ7, and (**H**) OK1.

The small molecule A485 was identified by virtual screening as a potent catalytic inhibitor of KAT3A/-3B [228,229] (Figure 16A). A485 demonstrates selectivity for not inhibiting the acetyltransferase activities of KAT2A, -2B, -6A, and -6B. However, at a higher concentration, A485 shows selectivity for bromodomain and extra-terminal bromodomain proteins and 150 nonepigenetic targets. It also displays binding to dopamine and serotonin transporters, along with a modest inhibition of Polo-like kinase 3. A485 treatment was shown to result in a dose-dependent decrease in H3K27ac. A485 selectively inhibits tumor proliferation, including several blood-related cancers and AR-positive PCa. Further, A485 inhibited tumor growth in castration-resistant PCa xenograft models; it also led to a decrease in the mRNA levels of MYC and the AR-dependent gene SLC45A3, and a reduction in MYC protein levels [228,229,230,231].

### 4.2. C646

The compound C646 was identified by in silico screening (Figure 16B) [230]. C646 has been tested in several cell lines and has also been shown to modulate the antibacterial properties of macrophages and inflammation [230,231,232]. KAT3A and -3B were highly expressed in five gastric cancer (GC) cell lines (SGC-7901, MKN45, MGC-803, BGC-823, and KATO III) compared with a normal human gastric epithelial cell line (GES-1) [230,231,232]. C646, a selective inhibitor of KAT3A and -3B, inhibited cell viability and cell cycle progression and promoted apoptosis in all five GC cell lines. Except for the MGC-803 cell line, KAT3A mRNA levels in the other four GC cell lines were significantly higher than those in GES-1 cells. The protein expression levels of KAT3A and -3B in the five GC cell lines were significantly higher than those in the GES-1 cells. Finally, C646 treatment significantly reduced the levels of histone H3 acetylation in both GC cells and normal gastric epithelial cells [230,231].

### 4.3. NiCur

The activation of p53-mediated apoptosis controls the growth of tumors and can trigger a massive loss of normal cells as side effects [55,194,223]. Structurally modified C5-curcumin analogs (both acyclic and cyclic) and a NiCur were previously demonstrated to exhibit anti-inflammatory, antiangiogenic, and anticancer activities. Cell-based screening assay revealed that NiCur was most potent in inhibiting KAT3A acetylation activity [223]. Molecular docking and in vitro acetylation assay revealed that NiCur binds to the active sites of KAT3A and not KAT2B. Additionally, under conditions of DNA damage, NiCur inhibited the levels of p53K382ac and the occupation of p53 on the *CDKN1A* promoter. Furthermore, NiCur inhibited apoptosis in normal gastric epithelial cells. Collectively, these data demonstrate that the inhibition of KAT3A epigenetic functions by NiCur can inhibit p53 toxicity in normal cells [55,194,223].

In summary, depending upon the biological context, the inhibition of KAT3A/-3B-mediated acetylation functions can block the growth of cancer cells and antagonize p53 activation. These data underline the broader implications of small molecule inhibitors, which could be developed further into a highly selective KAT inhibitor. Virtual screening of ligands has become a more effective tool to screen many compound libraries rapidly. It has the potential to address the underlying target-based mechanisms and structure-guided design of new analogs. In the future, chemically conjugating the inhibitors of KAT3A/-3B enzymatic activity with KAT3A/-3B bromodomain could entirely block the acetylation-based functions of KAT3A/-3B. These studies lay the foundation for developing high-affinity KAT3A modulators that could serve as a valuable mechanistic tool for transforming cellular fate by reprogramming the epigenetic landscape.

### 4.4. Structural Analysis of Ligands Binding to KAT3A/-3B Acetyltransferase Domain

**Figure 17:**
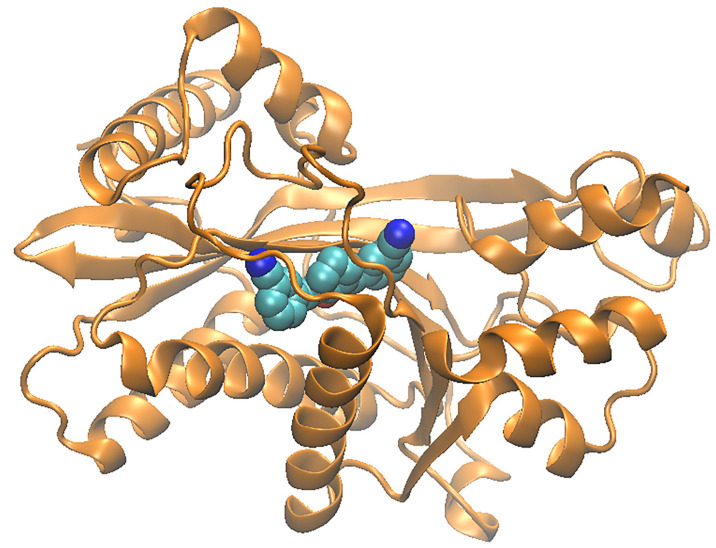
The ligand NiCur docked into the active site of KAT3A. The golden brown color indicates the acetyltransferase domain of KAT3A and the blue and metallic grey colors indicate the ligand, NiCur.

Several crystal structures of KAT3B bound to ligand are available in the PDB. The structures discussed here include PDB IDs 3biy, 4bhw, 5kj2, 6pf1, 6pgu, 6v8k, 6v8n, and 6v90, which bind ligands 01K [233], 6TF/A485 [228], OJ7 [234], OK7 [71,234], QS4 [71], QS1 [71], and QSD [71], respectively, as well as the docked pose of the ligand NiCur, developed in our laboratories. Note that the ligand labeled 6TF in the 5kj2 structure is A485 from the discussion above. The structures of ligands OK7 (Figure 16D), QS1 and QSD (Figure 16E), QS4 (Figure 16F), OJ7 (Figure 16G), and 01K (Figure 16H) are shown, respectively. The docked structure of our ligand NiCur docked to KAT3A is shown in Figure 17. The ligand-p300 structure from the PDB and the models of our compounds were analyzed for ligand-protein contacts. Contacts were defined as mutually proximal heavy atom pairs (e.g., atom L on the ligand is closest to atom P on the protein, and atom P is also the closest to atom L) [235]. Table 3 lists the closest contact distances between the various ligands. The residues that are parts of HX1 and HX2 are also marked. Note that KAT3B structures mentioned as PDB IDs 3biy and 4bhw bind to the same ligand (01K).

The binding patterns of ligands 01K, QS4, and QSD were relatively similar, despite the significant differences in their structures. Note that 01K was much larger than the other two. The ligands 6TF (i.e., A485), OK7, and QS4 seemed to belong to different families. Finally, the contact pattern of ligand OJ7 set it apart from the other ligands, since most of its contacts were with residues with which no other ligand was in contact, and it only shared a few contact residues with OK7 and QS4. It can be hypothesized that its action could be allosteric. Our ligand NiCur had relatively fewer contacts; most were common with the contacts of the first ligand family. If two ligands have the same binding affinity, but one of them have fewer contacts, the chances of that ligand being more specific to its target are likely to be higher.

## 5. Material and Methods

### Amino Acid Homology Alignment of KATs

KAT sequences were extracted from the human KAT-containing proteins KAT1 (NP003633.2), KAT2A (NP066564.2), KAT2B (NP003875.3), KAT3A (NP004371.2), KAT3B (NP001420.2), KAT4 (EAX05294.1), KAT6A (NP006757.2), KAT6B (NP001357065.1), KAT7 (NP008998.1), KAT8 (NP115564.2), KAT9 (NP060561.3), KAT13A (NP003734.3), KAT13B (NP858045.1), and KAT13C (NP001308636.1). An EMBL sequence analysis was then used to perform a multiple sequence alignment using the Clustal Omega program tool [142,143]. The alignment was imported to the program Jalview [236].

## 6. Discussion and Future Perspectives

The past three decades of research to uncover the functions of KATs have gained momentum with parallel improvements in genomic and proteomic technologies. The increasing knowledge of pathways regulated by acetylation and deacetylation has facilitated a better understanding of disease pathogenesis and identification of new therapeutic targets. However, future challenges are geared toward understanding the basis for tissue specificity by KATs, epigenetic signatures on chromatin-associated protein, and a molecular basis for selectivity in forming molecular complexes to drive gene transcription.

On the chromatin landscape, acetylation is widespread across all the histone proteins, including H2A, H2B, H3, and H4, as well as H1 [57,151,152]. The site-specific acetylation on chromatin is a marker of nucleosomal relaxation and gene activation [6,27,153]. Acetylation could enhance stability, cellular localization, and transcriptional activities of transcription factors [35,38,39]. X-ray crystallographic studies have provided three-dimensional insights into the active sites and cofactor binding sites of KATs [28,29]. Additionally, biochemical investigations using mutagenesis have confirmed the critical residues for acetyl-CoA binding and substrate binding. The development of antibodies to acetylated lysine was instrumental in understanding the cellular interactions by immunoprecipitation and localization by fluorescence-based microscopy [111]. ChIP-based techniques elucidated the dynamics of acetylation-dependent and -independent molecular interactions on the gene promoter. For structural and biochemical investigations, there are often limitations with the amount of protein and the level of purification. Cell-based assays can be time-consuming and expensive. In these situations, protein modeling and virtual screening using bioinformatics tools becomes essential.

The epigenetics field has expanded from chromatin biology to gene regulation after discovering acetylation on two crucial proteins: p53 and HIV Tat [43,44]. Acetylated proteins were noted from the nucleus to mitochondria, involving at least 2000 acetylated proteins. Together, these data implicated the role of acetylation in metabolism and cellular homeostasis [38,39]. Studies using cellular models of p53 have identified a multitier complexity in transcriptional regulation, driven by stress-induced epigenetic modifications and molecular interactions [111]. Acetylation of p53 is readily detectable using mass spectrometry, mutagenesis, and antibody-based approaches; however, probing the molecular basis of interactions with multiple domain-containing cofactors remains challenging. Additionally, identifying the determinants by which p53 exhibits promoter selectivity in many cell types will be challenging. The CTD of p53 serves as a recruiting platform for many cofactors with equal propensities to bind the N-terminal activation domains of p53. The role of the CTD concerning the oligomerization domain is not clearly understood. The acetylation of HIV Tat by KAT3B—leading to the bromodomain-mediated recruitment of KAT2B—is central to HIV replication. Blocking the ability of HIV Tat to recruit and exploit the system of human transcription will be a target for newer generations of anti-HIV drug discovery programs [149]. Targeting epigenetic mechanisms offers the potential to overcome drug resistance to antimicrobial agents.

Most of the potential KAT inhibitors exhibit antioxidant activity, reactivity, instability, low potency, or selectivity between KAT subtypes. KATs are bisubstrate enzymes that catalyze reactions between acetyl coenzyme A and a lysine-containing peptide. A detailed characterization of the catalytic mechanism and the kinetics of small molecule inhibitors will significantly improve the selectivity of KAT modulators and provide validated starting points for further development. The kinetics that drives epigenetic modifications and the mechanistic underpinnings of molecular interactions are essential to elucidate the mechanisms of acetylation-mediated pathways. Chemical biology and gene editing-based approaches can address the questions of selectivity during gene regulation. Without perturbing gene expression levels, small molecules could target the active sites of endogenous KATs to differentiate the role of acetylation versus molecular interactions during the process of gene activation. Gene-editing technologies (such as transcription activator-like effector nucleases and CRISPR) could provide more answers about how genes are regulated compared to transient expression systems. Overall, these efforts should provide a starting point for advancing the development of therapeutic agents.

## 7. Conclusions

The ability of the amino acid lysine to undergo a multitude of epigenetic modifications is remarkable. The molecular bases of potential mutually exclusive modifications on lysine residue need to be understood in terms of tissue specificity, type of stress, and disease conditions. KAT-mediated lysine acetylation serves as a major epigenetic mark that plays a pivotal role in orchestrating many biological processes, including growth, development, and cellular responses to external and internal changes. The abnormal functions of KATs—due to mutations or deregulated stoichiometry within molecular complexes—impact human health and diseases unambiguously. The intricacies in acetylation-mediated molecular events arise from the presence of myriad targets and multiple acetylation sites on the same target. Furthermore, the mechanistic basis for selectively recruiting bromodomains versus KDACs to the acetylation site is not clear. It is still not fully clear what drives the recruitment of KAT complexes to the chromatin of the target promoter. Although we have come a long way in understanding the functions of KATs, these data also underline a need to investigate the functions of KATs using innovative approaches in order to better understand the presence of disease-specific epigenetic signatures. Finally, it is also clear that there must be a combination of sensitive tools to investigate the dynamics of KATs in normal and pathological situations.

## Figures and Tables

**Table 1:** Chromosomal and cellular location of human KATs.

Name	Generic Name	Chromosomal Location	Number of Amino Acids	Cellular Location
KAT1	HAT1	2	419	Nucleus
KAT2A	GCN5	17	837	Nucleus
KAT2B	PCAF	3	832	Nucleus
KAT3A	CBP	16	2404	Nucleus
KAT3B	p300	22	2414	Nucleus
KAT4	TAF1	X	1893	Nucleus
KAT5	TIP60	11	546	Nucleus
KAT6A	MYST3	8	2004	Nucleus and Cytosol
KAT6B	MYST4	10	2073	Nucleus
KAT7	MYST2	17	611	Nucleus
KAT8	MYST1	16	458	Nucleus
KAT9	ELP3	8	547	Nucleus
KAT12	TFIIIC90	9	822	Nucleus
KAT13A	NCOA1	2	1399	Nucleus, Plasma Membrane, and Cytosol
KAT13B	NCOA3	20	1415	Nucleus and Cytosol
KAT13C	NCOA2	8	1464	Nucleus
KAT13D	CLOCK	4	846	Nucleus

**Table 2:** Tissue distribution and cancer specificity of KATs.

Name	Tissue	Cancer Type	References
KAT1	Appendix, bone marrow, lymph node, tonsil, nasopharynx, esophagus, stomach, duodenum, small intestine, colon, rectum, urinary bladder, testis, epididymis, vagina, cervix, uterine, endometrium, placenta, skin	Liver, ovarian, cervical, skin, testis	[59,60,61,62]
KAT2A	Skin, spleen, cerebral cortex, parathyroid	Renal, colorectal, melanoma, testis, thyroid	[48,62,63,64,65,66,67]
KAT2B	Cerebellum, thyroid, salivary, stomach, urinary bladder, placenta	Glioma, thyroid, melanoma	[48,62,68,69]
KAT3A	Cerebellum, thyroid, nasopharynx, gallbladder, oral mucosa, esophagus, small intestines, colon, rectum, urinary bladder, testis, fallopian tubes, vagina, cervix, uterine, endometrium, placenta, skin	Renal, thyroid, lung, head, neck, testis, breast	[62,70,71,72,73,74,75]
KAT3B	Cerebral cortex, parathyroid, adrenal, bone marrow, esophagus, colon, rectum, placenta, skin	Renal, thyroid, carcinoid, stomach, renal, head, neck	[62,70,71,72,73,74,75,76,77]
KAT4	Cerebellum, thyroid, salivary, stomach, urinary bladder, placenta, hippocampus, caudate, adrenal, appendix, tonsil, skeletal muscle, lung, nasopharynx, bronchus, gallbladder, esophagus, duodenum, small intestines, colon, rectum, kidney, testis, epididymis, fallopian tube, vagina, cervix, uterine, endometrium, ovary, placenta, soft tissues, skin, hippocampus, heart muscle, skeletal muscle, lung, bronchus, seminal vesicle, breast	Lung, glioma, thyroid, lymphoma, pancreatic, carcinoids	[62,78,79,80]
KAT5	Cerebral cortex, parathyroid, adrenal, caudate, cerebellum, thyroid, nasopharynx, colon, rectum, placenta, stomach, duodenum, ovary, cervix, uterine, oral mucosa, gallbladder	Renal, melanoma, testis, lymphoma	[62,81,82,83,84,85,86,87,88,89,90,91,92,93,94,95,96]
KAT6A	Cerebral cortex, caudate, cerebellum	Glioma, thyroid, carcinoid	[62,89,90,91,92,93,94,95,96]
KAT6B	Cerebellum, thyroid, salivary, stomach, urinary bladder, placenta, hippocampus, caudate, adrenal, appendix, tonsil, skeletal muscle, lung, nasopharynx, bronchus, gallbladder, esophagus, duodenum, small intestines, colon, rectum, kidney, testis, epididymis, fallopian tube, vagina, cervix, uterine, endometrium, ovary, placenta, soft tissues, skin	Renal, glioma, thyroid, lung, head, skin, neck	[62,96,97]
KAT7	Testis	Glioma, thyroid, melanoma	[62,98,99,100,101]
KAT8	-	Lung, breast, endometrial	[62,102,103,104,105,106]
KAT9	Appendix, duodenum, small intestine, colon, rectum, kidney, urinary bladder, prostate, endometrium, placenta	Renal, colorectal, thyroid, prostate, liver	[62,107,108,109]
KAT13A	Cerebral cortex, hippocampus, cerebellum, thyroid, parathyroid, adrenal, lymph node, nasopharynx, bronchus, gallbladder, pancreas, oral mucosa, esophagus, stomach, duodenum, small intestines, colon, rectum, kidney, urinary bladder, testis, fallopian tube, breast, vagina, placenta	Thyroid, carcinoid, head, neck	[62,110,111,112,113,114,115,116,117,118,119,120,121]
KAT13B	Cerebral cortex, hippocampus, cerebellum, thyroid, parathyroid, adrenal, lymph node, nasopharynx, bronchus, gallbladder, pancreas, oral mucosa, esophagus, stomach, duodenum, small intestines, colon, rectum, kidney, urinary bladder, testis, fallopian tube, breast, vagina, placenta, soft tissue, skin	Thyroid, carcinoid, head, neck	[122,123,124,125,126,127]
KAT13D	Cerebral cortex, hippocampus, cerebellum, thyroid, parathyroid, adrenal, lymph node, nasopharynx, bronchus, gallbladder, pancreas, oral mucosa, esophagus, stomach, duodenum, small intestines, colon, rectum, kidney, urinary bladder, testis, fallopian tube, breast, vagina, placenta, soft tissue, skin	Thyroid, breast, cervical, head, neck	[62,128]

**Table 3:** Residue–ligand distances for residues in contact with the ligand.

PDB id:			3biy	4bhw	5kj2	6pf1	6pgu	6v8k	6v8n	6v90	
Ligand:			01K	01K	6TF	OJ7	OK7	QS4	QS1	QSD	NiCur
1374	PHE				2.99						
1379	GLN							3.68			
1394	TYR						3.52	3.23			
1395	ILE										2.95
1396	SER		3.34	3.17				2.78			
1397	TYR		3.22	2.49				3.23			
1398	LEU		2.91	2.9					3.21	3.24	
1399	ASP			3.35	3.32				3.25	3.1	
1400	SER		2.62	2.45	2.94				2.69	2.89	
1407	LYS	HX2		3.65							
1410	ARG	HX2	2.89	2.93							
1411	THR	HX2	2.68								
1414	TYR	HX2	3.47		3.37				3.7	3.86	
1434	HIS					3.33	3.18				
1435	ILE						3.08				
1436	TRP		2.96	2.78				2.87			3.06
1438	CYS		3.68	3.7							2.7
1440	PRO		3.24	3.51	3.07				3.87		
1443	GLY								3.41	3.53	
1444	ASP										
1446	TYR		3.68	3.44							2.92
1451	HIS				3.24				3.39	3.31	
1452	PRO										
1453	PRO										
1455	GLN				3.05				3.21	3.23	3.07
1456	LYS		3.48								
1457	ILE		2.91	2.69							
1458	PRO		3.17	3.37	3.09					3.89	3.34
1462	ARG	HX1	3.22	3.26	2.99						
1463	LEU	HX1	3.93	4.07						3.85	3.27
1466	TRP	HX1		2.78	2.9				3.19	3.33	3.8
1467	PHE	HX1	3.11	3.44							2.39
1486	ILE					3.25					
1489	GLN					3.21					
1490	ALA					3.06					
1495	LEU					3.4					
1501	LEU					3.32					
1502	PRO					3.25	3.11				
1505	GLU							3.53			
1507	ASP					2.87	2.8				
1509	TRP					3.53					
1591	HIS							3.29			
1595	PHE					3.56					
1596	PHE					3.2					
1597	VAL					3.79					

## Data Availability

Not applicable.

## Supplementary Materials

The following are available online at https://www.mdpi.com/2218-273X/11/3/455/s1, Figure S1: Multiple sequence alignment of the 16 human KATs discussed in this paper, displayed with the program Jalview. The phylogenetic tree shown in Figure 6B was based on this alignment. Table S1: Biochemical details of the KATs, including their generic name, chromosomal location, number of amino acids, and cellular locations. Table S2: Human body distribution and expression of KATs in tissues and respective cancers. Table S3: The modeling of KAT3A/-3B and its ligands reveals the residue-ligand distances for residues in contact with the ligand.
