# Supplementary material for: The Biological Significance of Targeting Acetylation-Mediated Gene Regulation for Designing New Mechanistic Tools and Potential Therapeutics
Source: Biomolecules. 2021 Mar 18;11(3):455. doi: 10.3390/biom11030455 (PMC8003229; doi:10.3390/biom11030455)
Supplement: Supplementary file 1 [file biomolecules-11-00455-s001.pdf]

Supplementary Data

The Biological Significance of Targeting Acetylation-mediated Gene Regulation for Designing New Mechanistic Tools and Potential Therapeutics

Chenise O'Garro[1#], Loveth Igbineweka[1#], Zonaira Ali[1], Mihaly Mezei[2], and Shiraz Mujtaba[1*]

The EMBL sequence analysis was then used to perform multiple sequence alignment using the Clustal Omega program tool' is missing the word tool after sequence analysis.

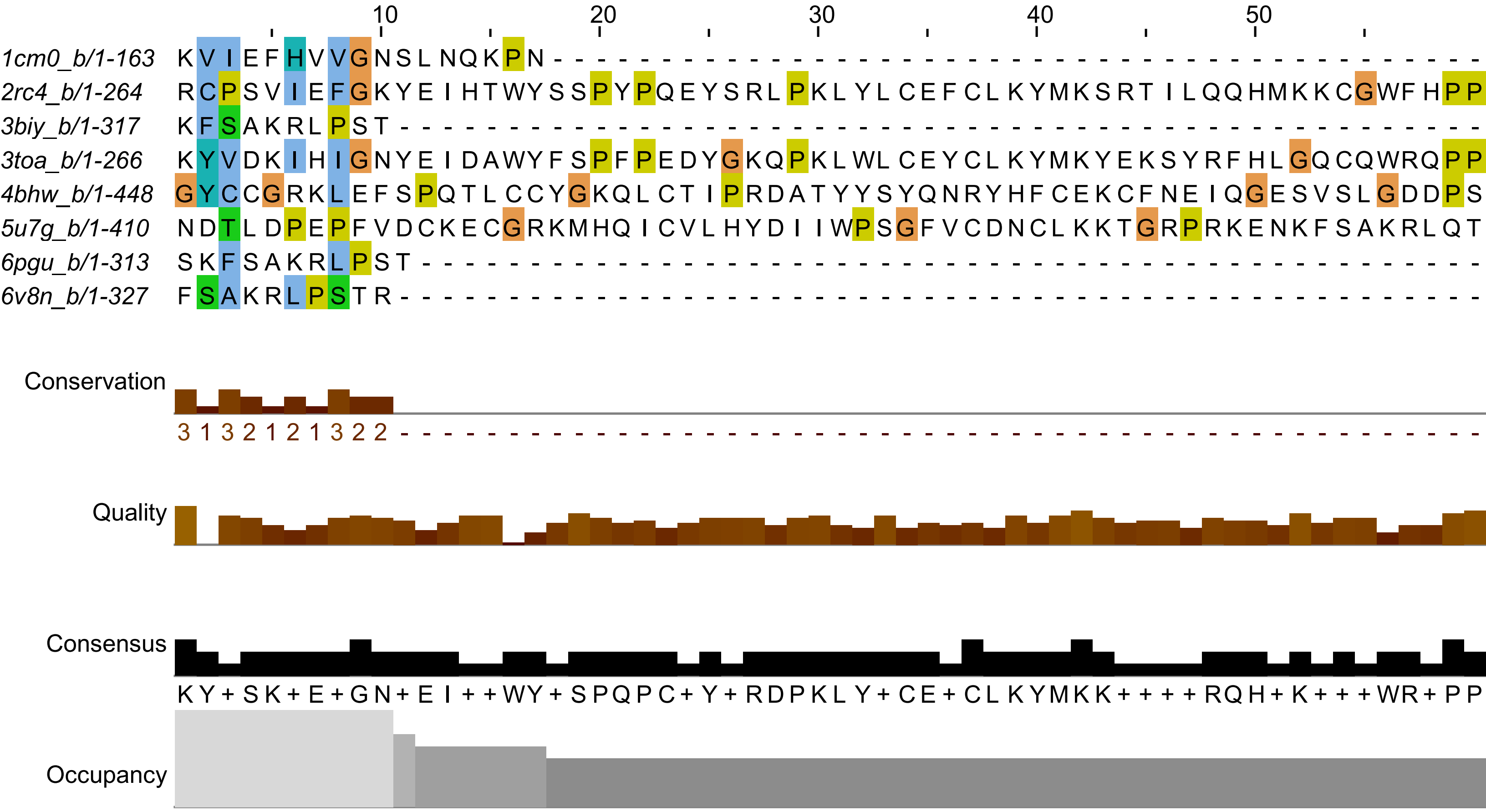
10 20 30 40 50
1cm0_b/1-163 KVIEFHVVGNSLNQKPN
2rc4_b/1-264 RCPSVIEFGKYEIHTWYSSPYPQEYSRLPKLYLCEFCLKYMKSRTILQQHMKKCGWFHPP
3biy_b/1-317 KFSAKRLPST
3toa_b/1-266 KYVDKIHIGNYEIDAWYFSPFPEDYGKQPKLWLCEYCLKYMKYEKSYRFHLGQCQWRQPP
4bhw_b/1-448 GYCCGRKLEFSPQTLCCYGKQLCTIPRDATYYSYQNRYHFCEKCFNEIQGESVSLGDDPS
5u7g_b/1-410 NDTLDPEPFVDCKECGRKMHQICVLHYDIIWPSGFVCDNCLKKTGRPRKENKFSAKRLQT
6pgu_b/1-313 SKFSAKRLPST
6v8n_b/1-327 FSAKRLPSTR
Conservation
3 1 3 2 1 2 1 3 2 2
Quality
Consensus
KY+SK+E+GN+EI++WY+SPQPC+Y+RDPKLY+CE+CLKYMKK++++RQH+K+++WR+PP
Occupancy

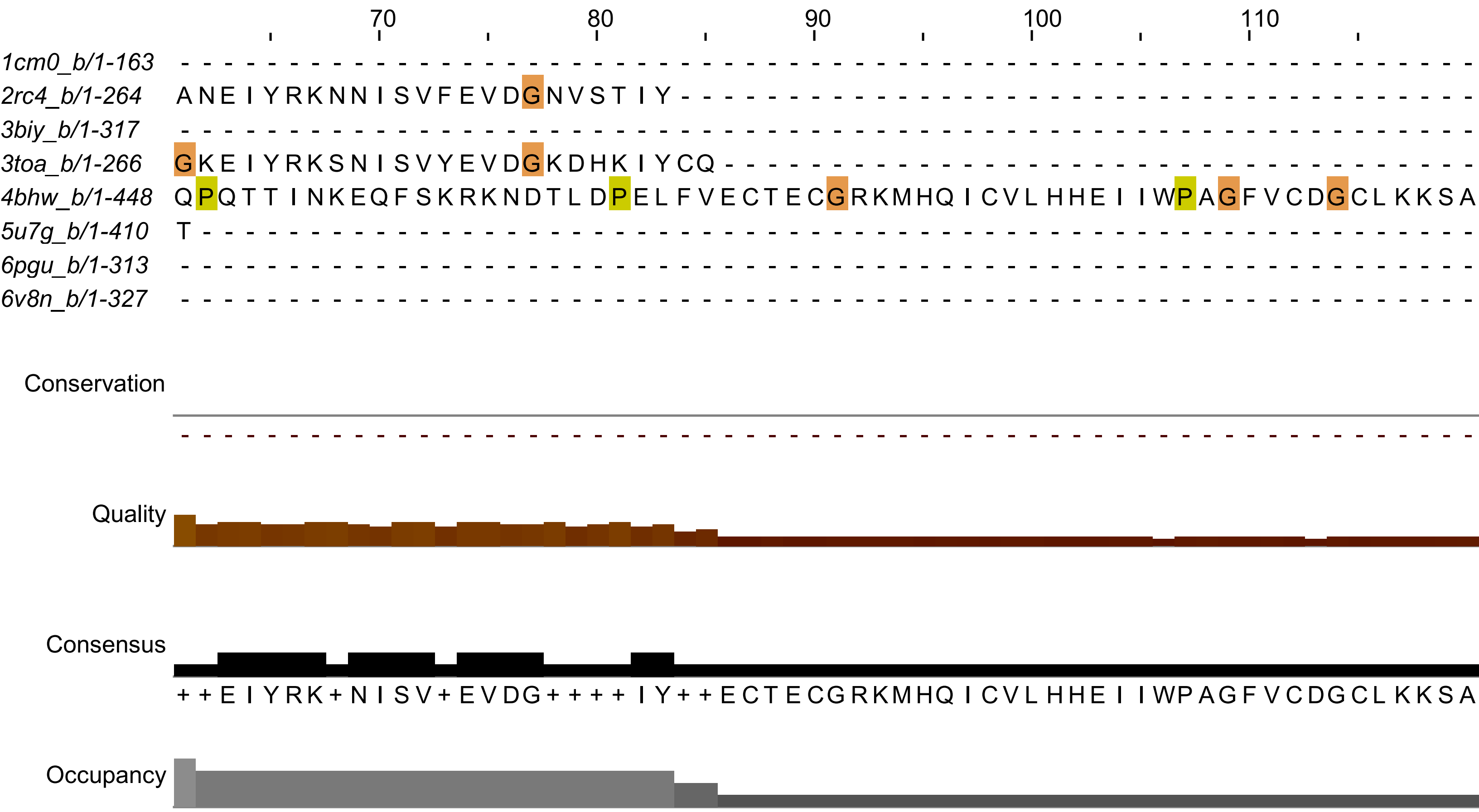
70 80 90 100 110
1cm0_b/1-163
2rc4_b/1-264 ANEIYRKNNISVFEVDGNVSTIY
3biy_b/1-317
3toa_b/1-266 GKEIYRKSNISVYEVDGKDHKIYCQ
4bhw_b/1-448 QPQTTINKEQFSKRKNDTLDPELFVECTECGRKMHQICVLHHEIIWPAGFVCDGCLKKSA
5u7g_b/1-410 T
6pgu_b/1-313
6v8n_b/1-327
Conservation
Quality
Consensus
++EIYRK+NISV+EVDG++++IY++ECTECGRKMHQICVLHHEIIWPAGFVCDGCLKKSA
Occupancy

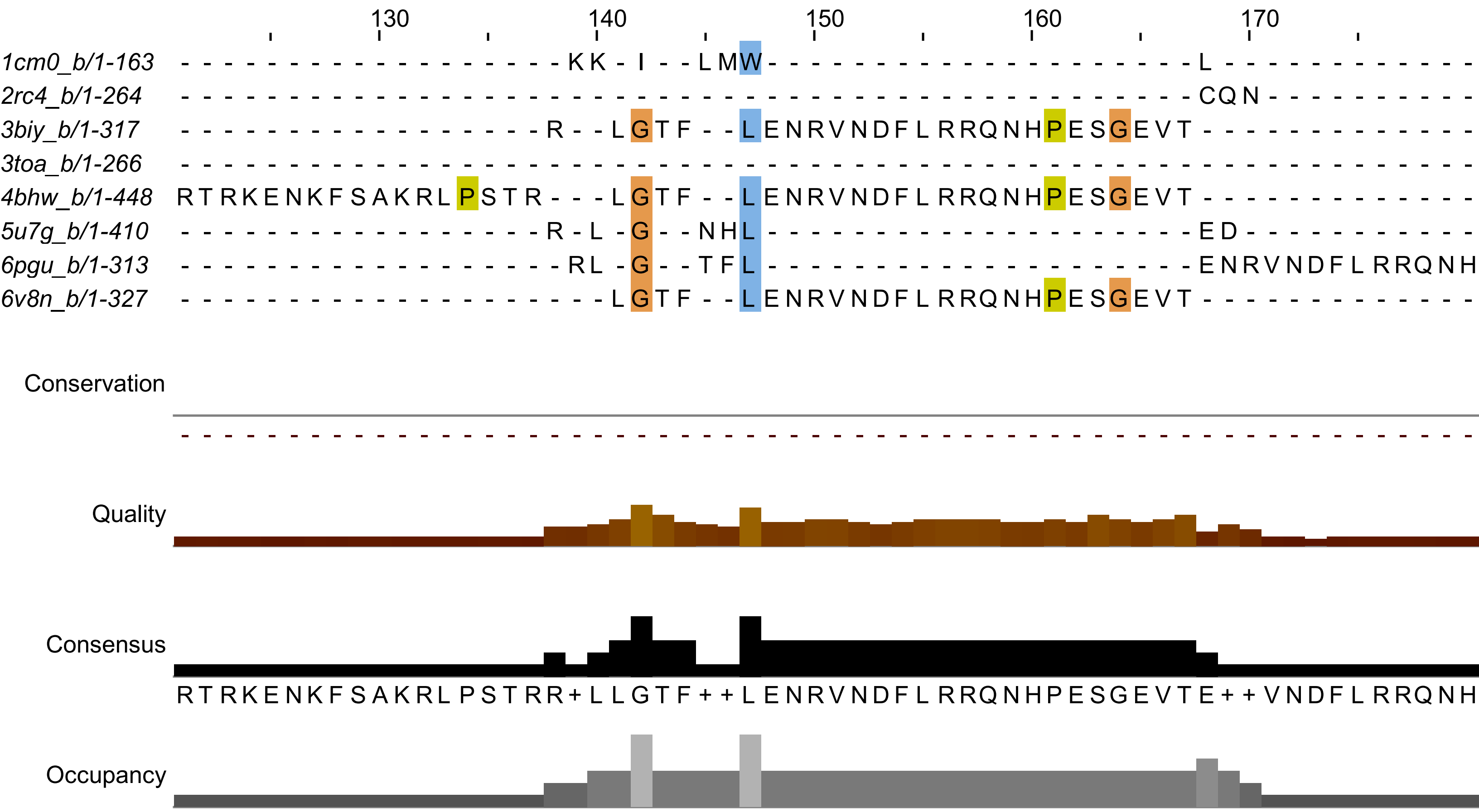
130
140
150
160
170
1cm0_b/1-163 - - - - - - - - - - - - - - - - - - K K - I - - L M W - L - - - - - - - - - - - -
2rc4_b/1-264 - C Q N - - - - - - - - - -
3biy_b/1-317 - - - - - - - - - - - - - - - - - R - - L G T F - - L E N R V N D F L R R Q N H P E S G E V T - - - - - - - - - - - - - -
3toa_b/1-266 -
4bhw_b/1-448 R T R K E N K F S A K R L P S T R - - - L G T F - - L E N R V N D F L R R Q N H P E S G E V T - - - - - - - - - - - - - -
5u7g_b/1-410 - - - - - - - - - - - - - - - - - R - L - G - - N H L - E D - - - - - - - - - - -
6pgu_b/1-313 - - - - - - - - - - - - - - - - - - R L - G - - T F L - E N R V N D F L R R Q N H
6v8n_b/1-327 - L G T F - - L E N R V N D F L R R Q N H P E S G E V T - - - - - - - - - - - - - -
Conservation
Quality
Consensus
R T R K E N K F S A K R L P S T R R + L L G T F + + L E N R V N D F L R R Q N H P E S G E V T E + + V N D F L R R Q N H
Occupancy

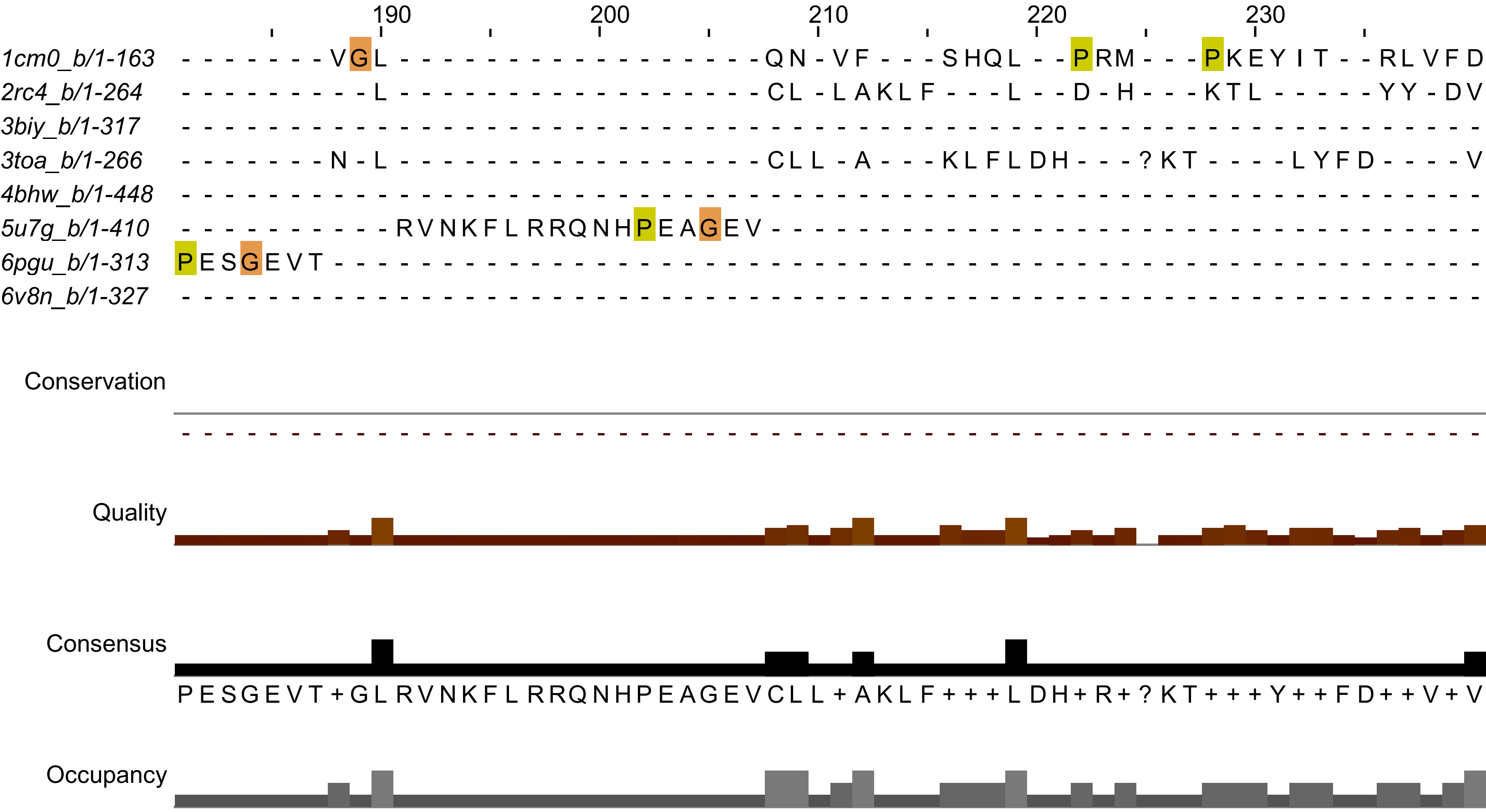
190
200
210
220
230
1cm0_b/1-163 - - - - - - - V G L - - - - - - - - - - - - - - - - - - Q N - V F - - - S H Q L - - P R M - - - P K E Y I T - - R L V F D
2rc4_b/1-264 - - - - - - - - - L - - - - - - - - - - - - - - - - - - C L - L A K L F - - - L - - D - H - - - K T L - - - - - Y Y - D V
3biy_b/1-317
3toa_b/1-266 - - - - - - - N - L - - - - - - - - - - - - - - - - - - C L L - A - - - K L F L D H - - - ? K T - - - - L Y F D - - - - V
4bhw_b/1-448
5u7g_b/1-410 - - - - - - - - - - R V N K F L R R Q N H P E A G E V
6pgu_b/1-313 P E S G E V T
6v8n_b/1-327
Conservation
Quality
Consensus
P E S G E V T + G L R V N K F L R R Q N H P E A G E V C L L + A K L F + + + L D H + R + ? K T + + + Y + + F D + + V + V
Occupancy

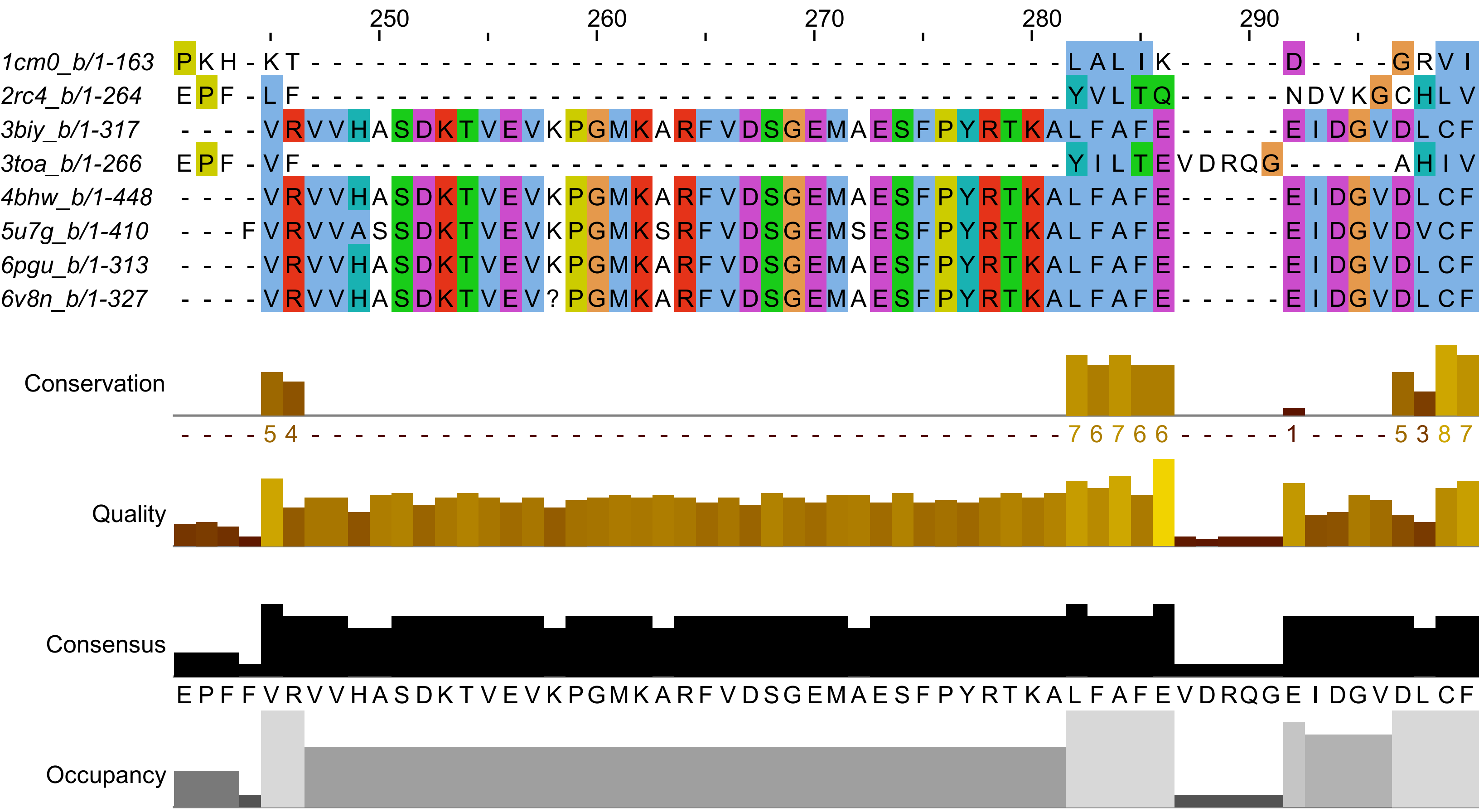

250 260 270 280 290
1cm0_b/1-163 PKH-KT-----------------------------------LALIK-----D----GRVI
2rc4_b/1-264 EPF-LF-----------------------------------YVLTQ-----NDVKGCHLV
3biy_b/1-317 ----VRVVHASDKTVEVKPGMKARFVDSGEMAESFPYRTKALFAFE-----EIDGVDLCF
3toa_b/1-266 EPF-VF-----------------------------------YILTEVDRQG-----AHIV
4bhw_b/1-448 ----VRVVHASDKTVEVKPGMKARFVDSGEMAESFPYRTKALFAFE-----EIDGVDLCF
5u7g_b/1-410 ---FVRVVASSDKTVEVKPGMKSRFVDSGEMSESFPYRTKALFAFE-----EIDGVDVCF
6pgu_b/1-313 ----VRVVHASDKTVEVKPGMKARFVDSGEMAESFPYRTKALFAFE-----EIDGVDLCF
6v8n_b/1-327 ----VRVVHASDKTVEV?PGMKARFVDSGEMAESFPYRTKALFAFE-----EIDGVDLCF
Conservation
----54-----------------------------------76766-----1----5387
Quality
Consensus
EPFFVRVVHASDKTVEVKPGMKARFVDSGEMAESFPYRTKALFAFEVDRQGEIDGVDLCF
Occupancy

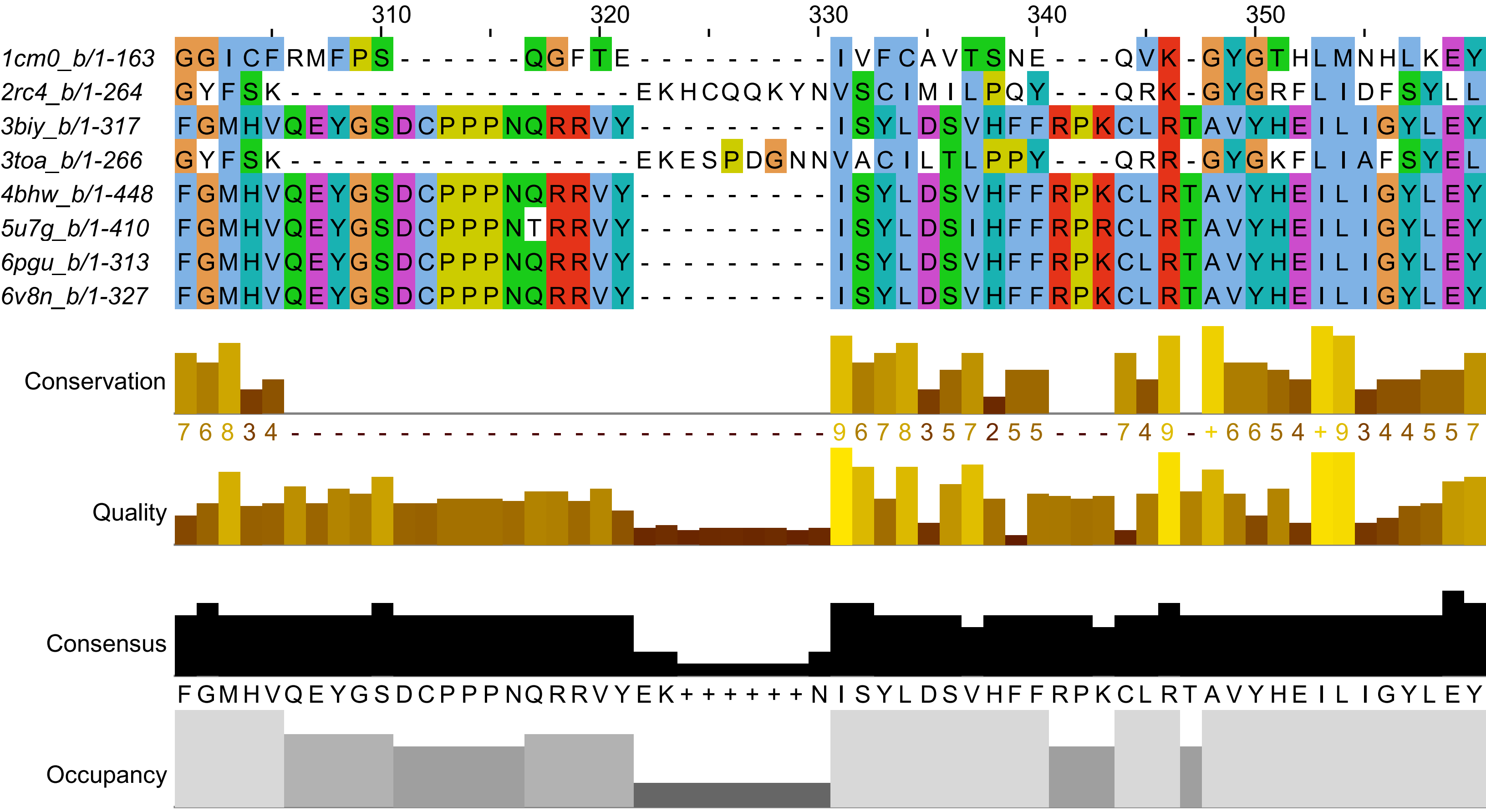

1cm0_b/1-163
2rc4_b/1-264
3biy_b/1-317
3toa_b/1-266
4bhw_b/1-448
5u7g_b/1-410
6pgu_b/1-313
6v8n_b/1-327
Conservation
Quality
Consensus
Occupancy

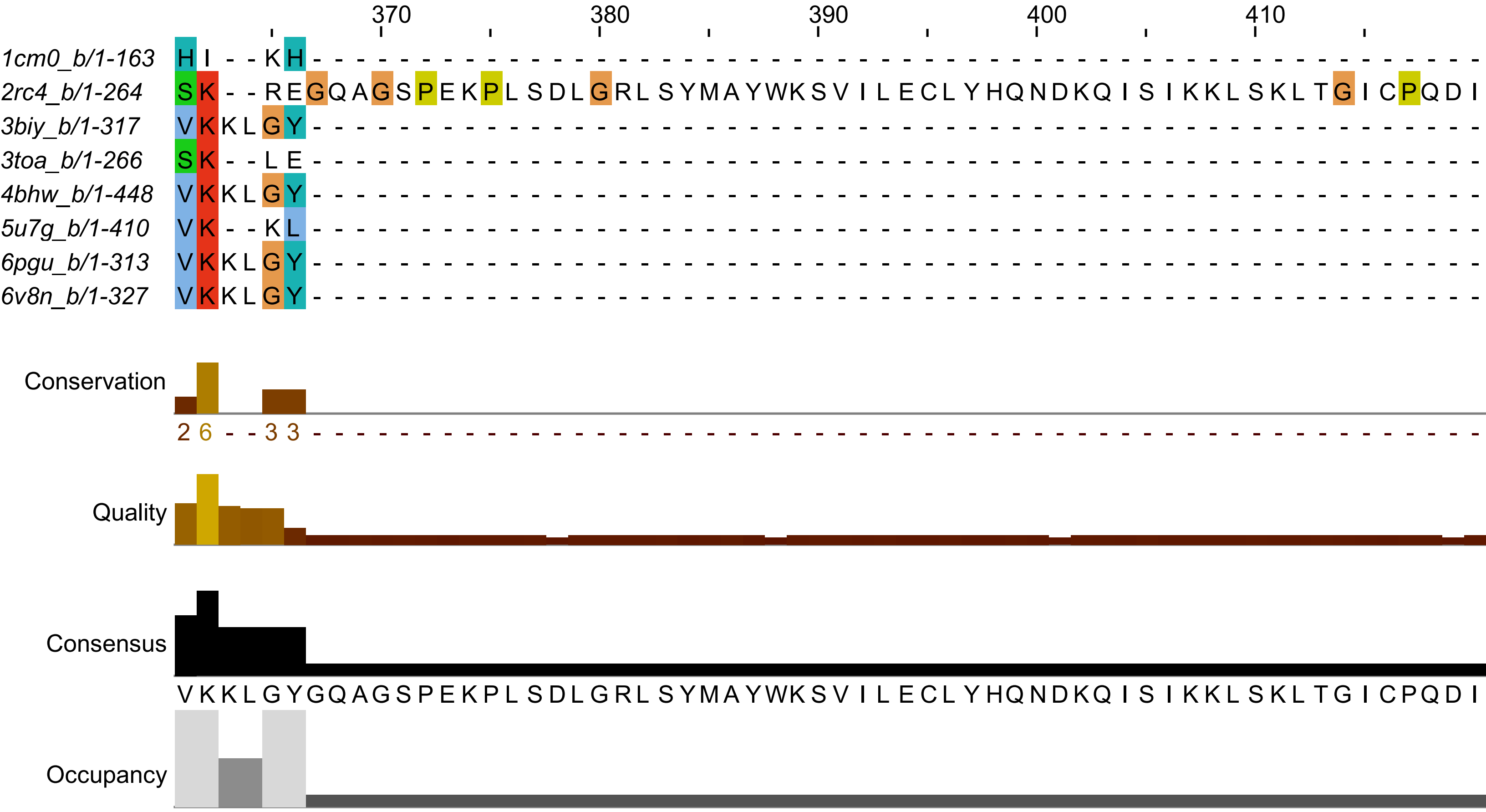

370
380
390
400
410
1cm0_b/1-163
2rc4_b/1-264
3biy_b/1-317
3toa_b/1-266
4bhw_b/1-448
5u7g_b/1-410
6pgu_b/1-313
6v8n_b/1-327
Conservation
Quality
Consensus
Occupancy
VKKLGYGQAGSPEKPLSDLGRLSYMAYWKSVILECLYHQNDKQISIKKLSKLTGICPQDI

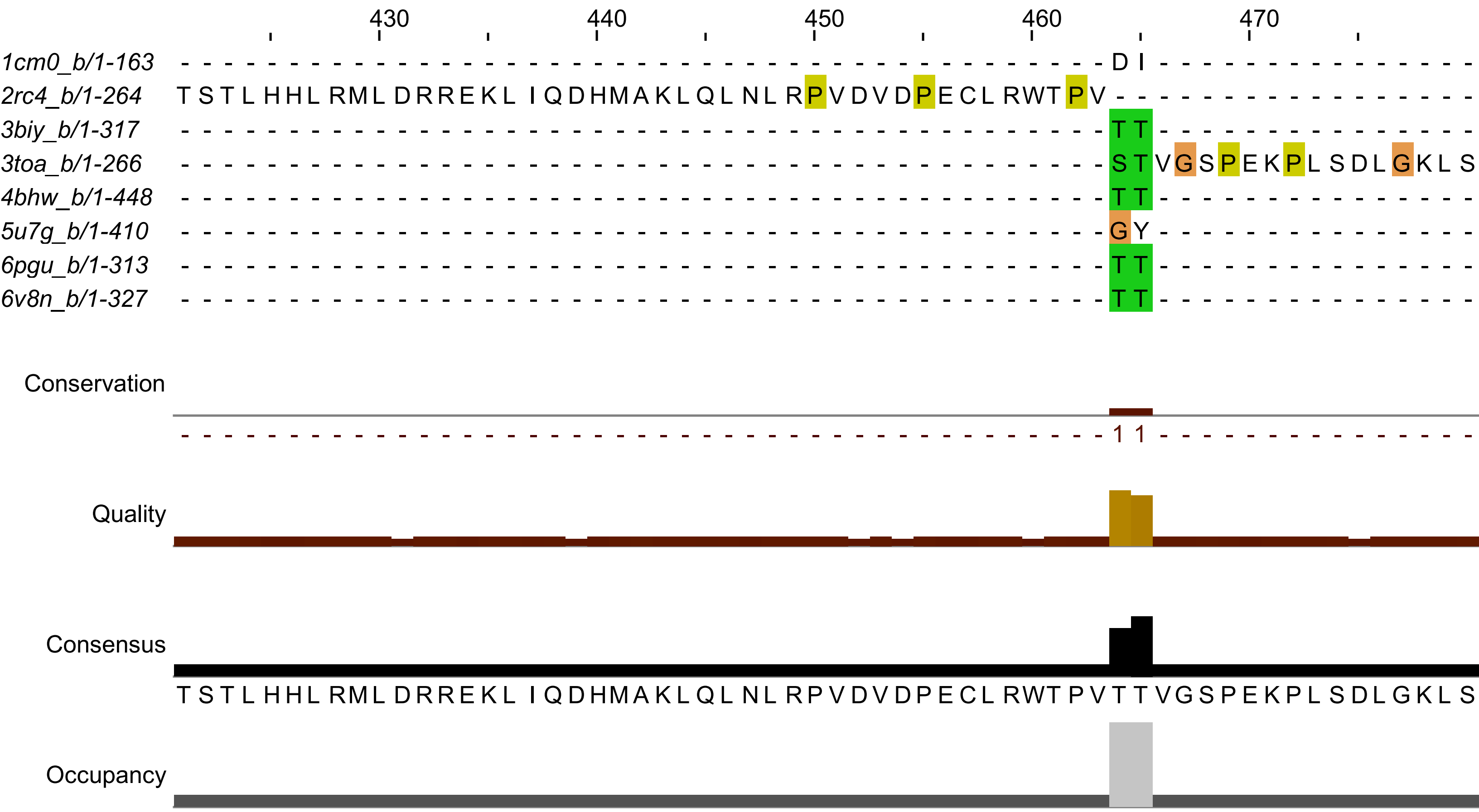
430
440
450
460
470
1cm0_b/1-163
2rc4_b/1-264
3biy_b/1-317
3toa_b/1-266
4bhw_b/1-448
5u7g_b/1-410
6pgu_b/1-313
6v8n_b/1-327
D I
T S T L H H L R M L D R R E K L I Q D H M A K L Q L N L R P V D V D P E C L R W T P V
T T
S T V G S P E K P L S D L G K L S
T T
G Y
T T
T T
Conservation
1 1
Quality
Consensus
T S T L H H L R M L D R R E K L I Q D H M A K L Q L N L R P V D V D P E C L R W T P V T T V G S P E K P L S D L G K L S
Occupancy

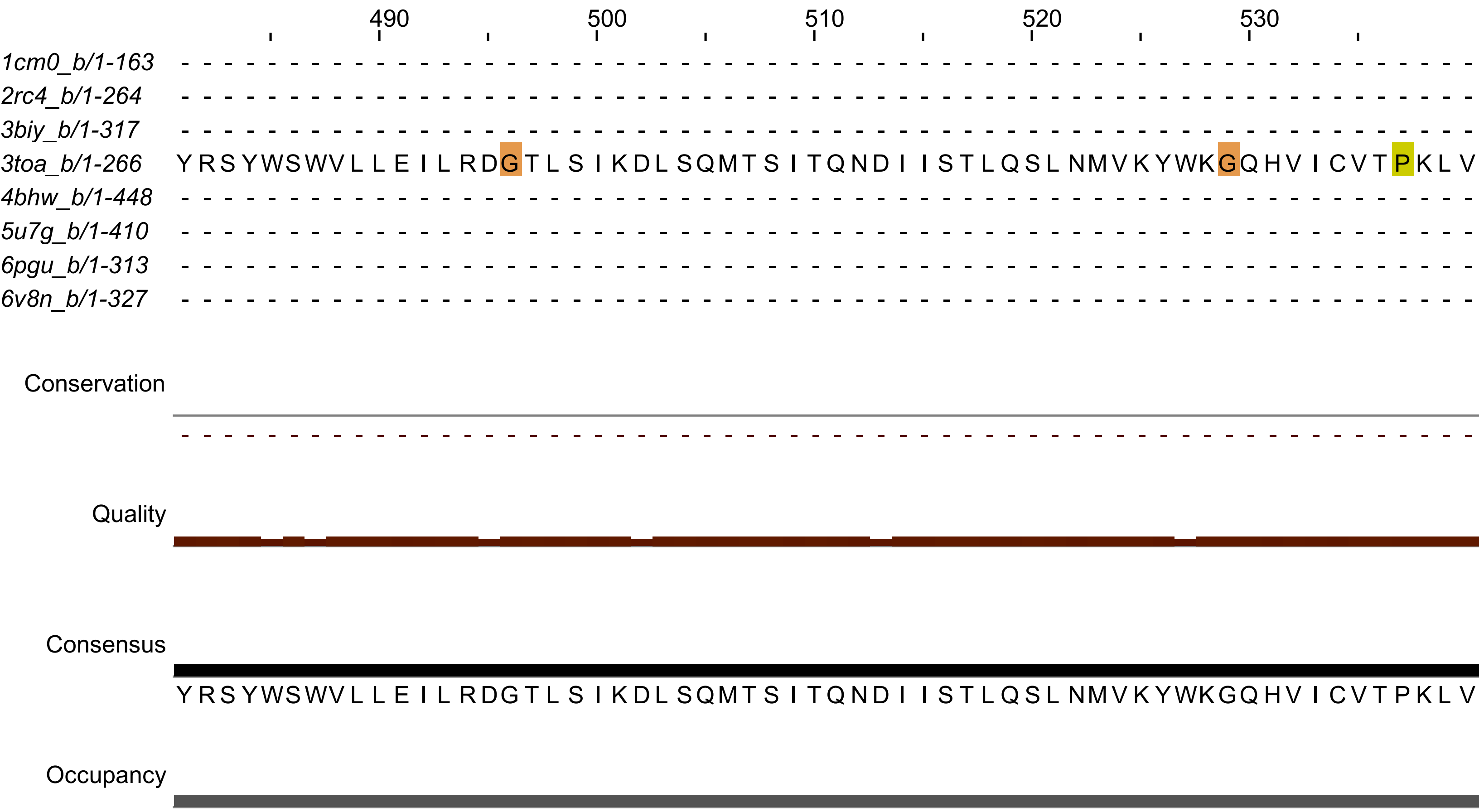

490
500
510
520
530
1cm0_b/1-163
2rc4_b/1-264
3biy_b/1-317
3toa_b/1-266
YRSYWSWVLLEILRDGTLSIKDLSQMTSITQNDIISTLQSLNMVKYWKGQHVICVTPKLV
4bhw_b/1-448
5u7g_b/1-410
6pgu_b/1-313
6v8n_b/1-327
Conservation
Quality
Consensus
YRSYWSWVLLEILRDGTLSIKDLSQMTSITQNDIISTLQSLNMVKYWKGQHVICVTPKLV
Occupancy

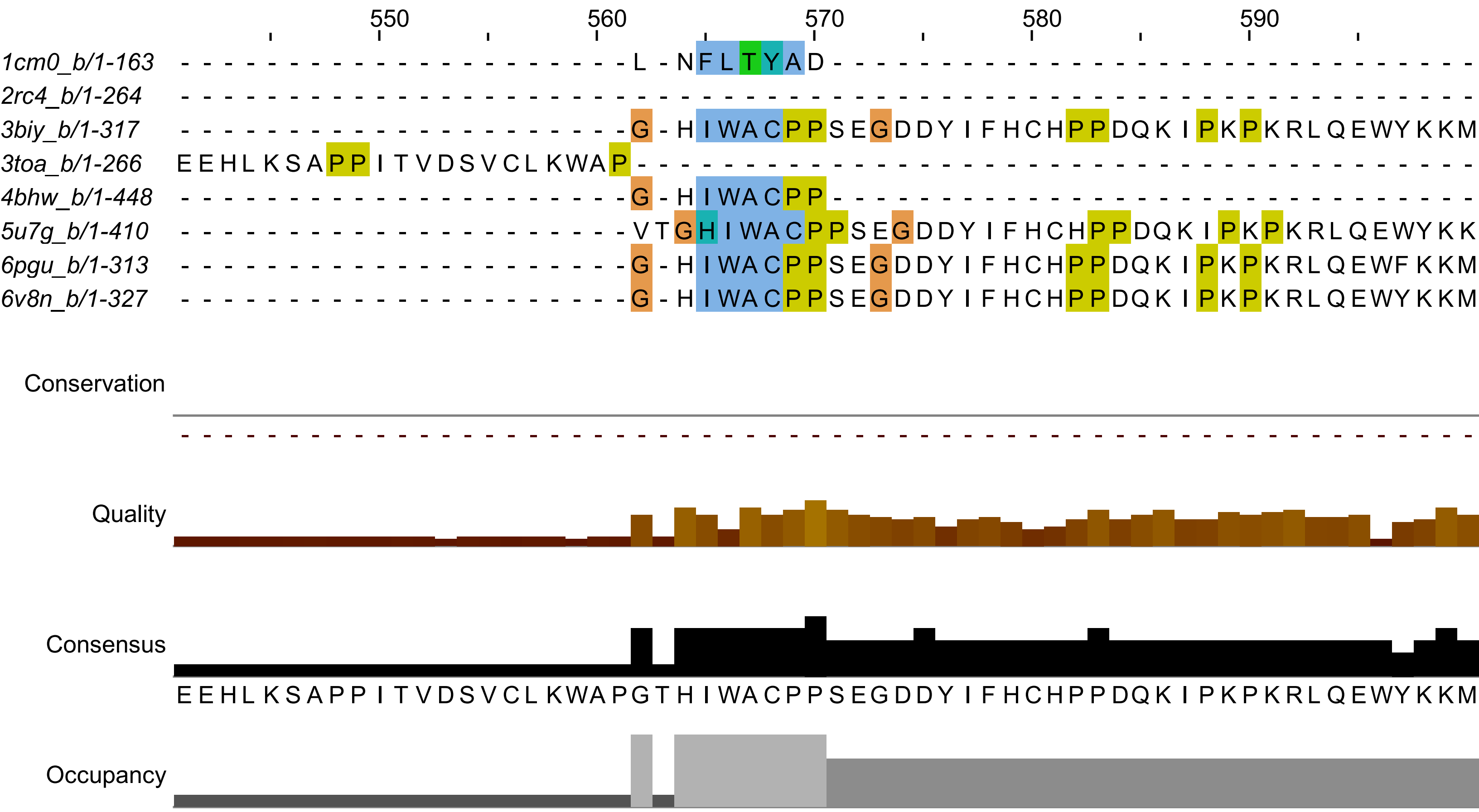
550 560 570 580 590
1cm0_b/1-163 - L - N F L T Y A D -
2rc4_b/1-264
3biy_b/1-317 - G - H I WA C P P S E G D D Y I F H C H P P D Q K I P K P K R L Q E WY K K M
3toa_b/1-266 E E H L K S A P P I T V D S V C L K WA P -
4bhw_b/1-448 - G - H I WA C P P -
5u7g_b/1-410 - V T G H I WA C P P S E G D D Y I F H C H P P D Q K I P K P K R L Q E WY K K
6pgu_b/1-313 - G - H I WA C P P S E G D D Y I F H C H P P D Q K I P K P K R L Q E WF K K M
6v8n_b/1-327 - G - H I WA C P P S E G D D Y I F H C H P P D Q K I P K P K R L Q E WY K K M
Conservation
Quality
Consensus
E E H L K S A P P I T V D S V C L K WA P G T H I WA C P P S E G D D Y I F H C H P P D Q K I P K P K R L Q E WY K K M
Occupancy

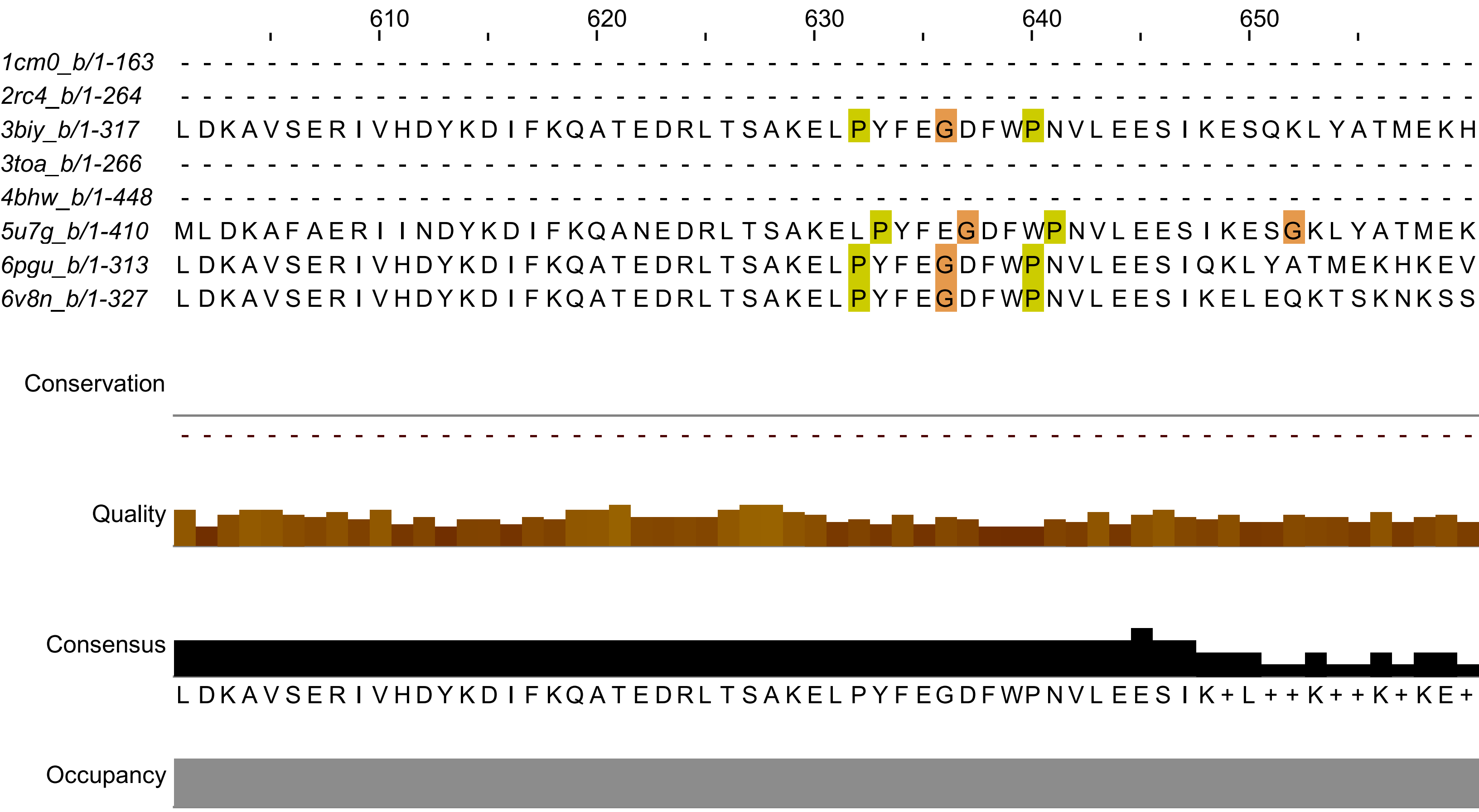
610 620 630 640 650
1cm0_b/1-163
2rc4_b/1-264
3biy_b/1-317 LDKAVSERIVHDYKDIFKQATEDRLTSAKELPYFEGDFWPNVLEESIKESQKLYATMEKH
3toa_b/1-266
4bhw_b/1-448
5u7g_b/1-410 MLDKAFAERIINDYKDIFKQANEDRLTSAKELPYFEGDFWPNVLEESIKESGKLYATMEK
6pgu_b/1-313 LDKAVSERIVHDYKDIFKQATEDRLTSAKELPYFEGDFWPNVLEESIQKLYATMEKHKEV
6v8n_b/1-327 LDKAVSERIVHDYKDIFKQATEDRLTSAKELPYFEGDFWPNVLEESIKELEQKTSKNKSS
Conservation
Quality
Consensus
LDKAVSERIVHDYKDIFKQATEDRLTSAKELPYFEGDFWPNVLEESIK+L++K++K+KE+
Occupancy

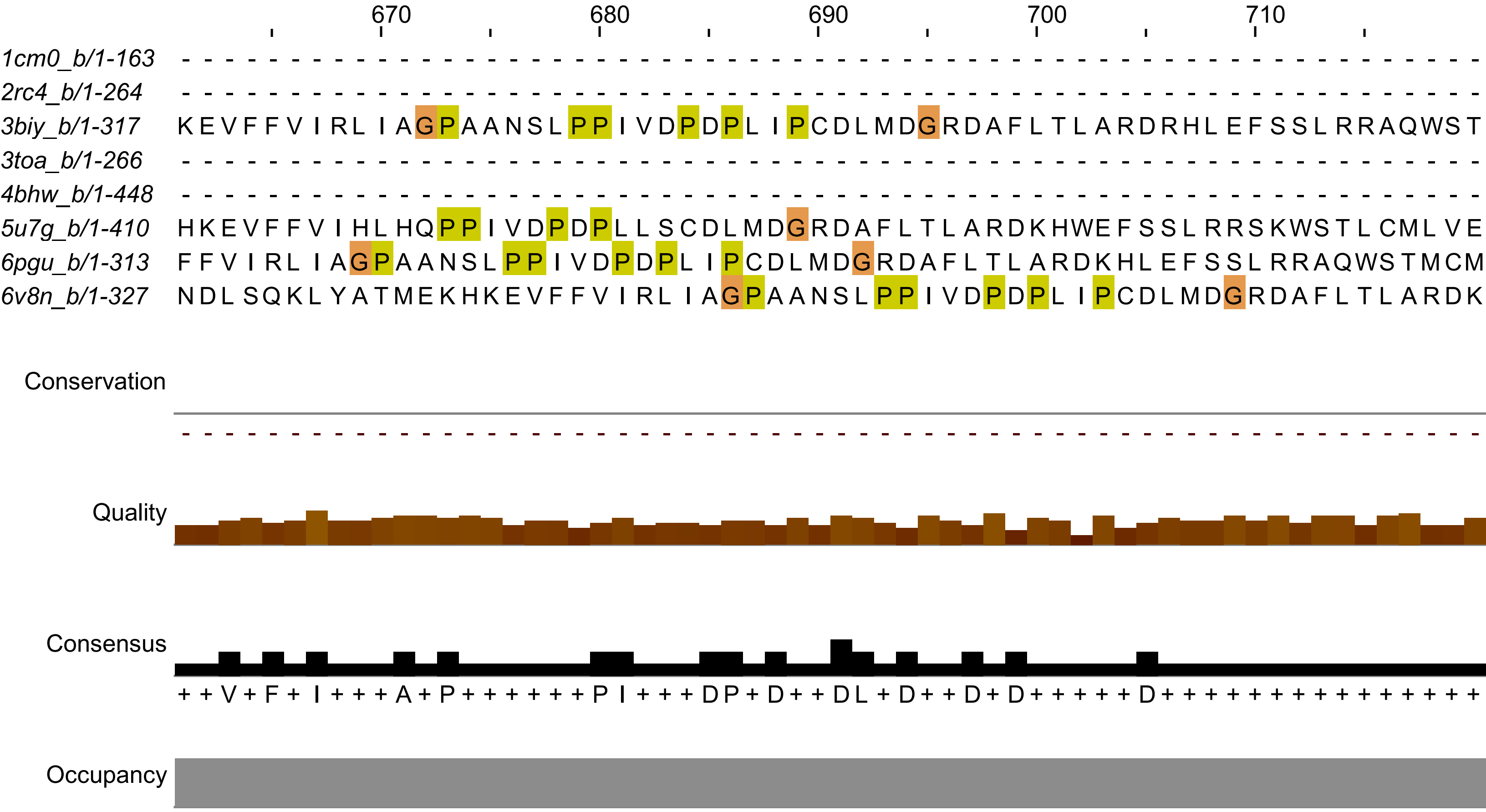

670
680
690
700
710
1cm0_b/1-163
2rc4_b/1-264
3biy_b/1-317 KEVFFVIRLIAGPAANSLPPIVDPDPLIPCDLMDGRDAFLTLARDRHLEFSSLRRAQWST
3toa_b/1-266
4bhw_b/1-448
5u7g_b/1-410 HKEVFFVIHLHQPPIVDPDPLLSCDLMDGRDAFLTLARDKHWEFSSLRRSKWSTLCMLVE
6pgu_b/1-313 FFVIRLIAGPAANSLPPIVDPDPLIPCDLMDGRDAFLTLARDKHLEFSSLRRAQWSTMCM
6v8n_b/1-327 NDLSQKLYATMEKHKEVFFVIRLIAGPAANSLPPIVDPDPLIPCDLMDGRDAFLTLARDK
Conservation
Quality
Consensus
++V+F+I+++A+P++++++PI+++DP+D++DL+D++D+D+++++D++++++++++++++++
Occupancy

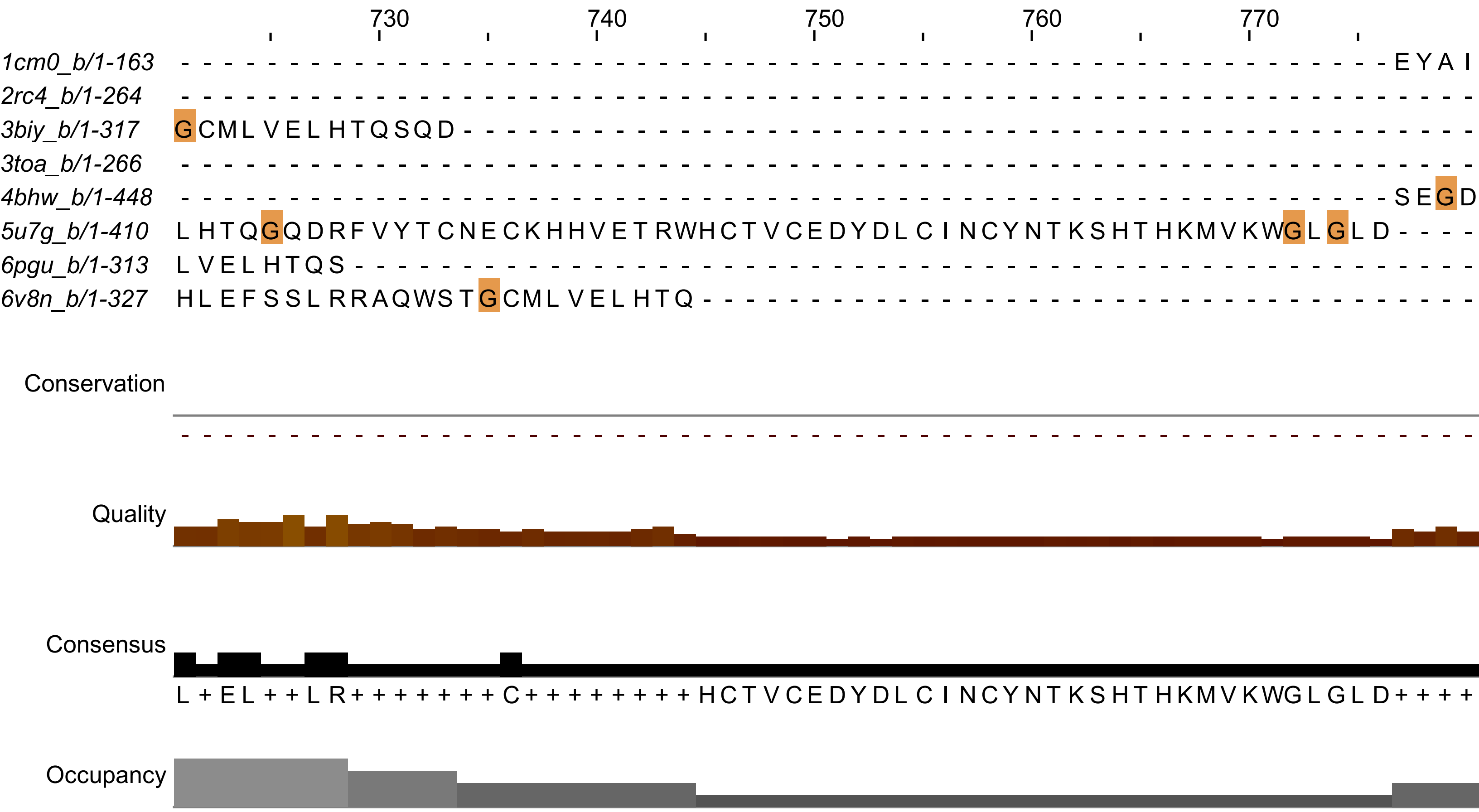
730 740 750 760 770
1cm0_b/1-163 EYAI
2rc4_b/1-264
3biy_b/1-317 GCMLVELHTQSQD
3toa_b/1-266
4bhw_b/1-448 SEGD
5u7g_b/1-410 LHTQGQDRFVYTCNECKHHVETRWHCTVCEDYDLCINCYNTKSHTHKMVKWGLGLD
6pgu_b/1-313 LVELHTQS
6v8n_b/1-327 HLEFSSLRRAQWSTGCMLVELHTQ
Conservation
Quality
Consensus
L+EL++LR+++++++C++++++++HCTVCEDYDLCINCYNTKSHTHKMVKWGLGLD++++
Occupancy

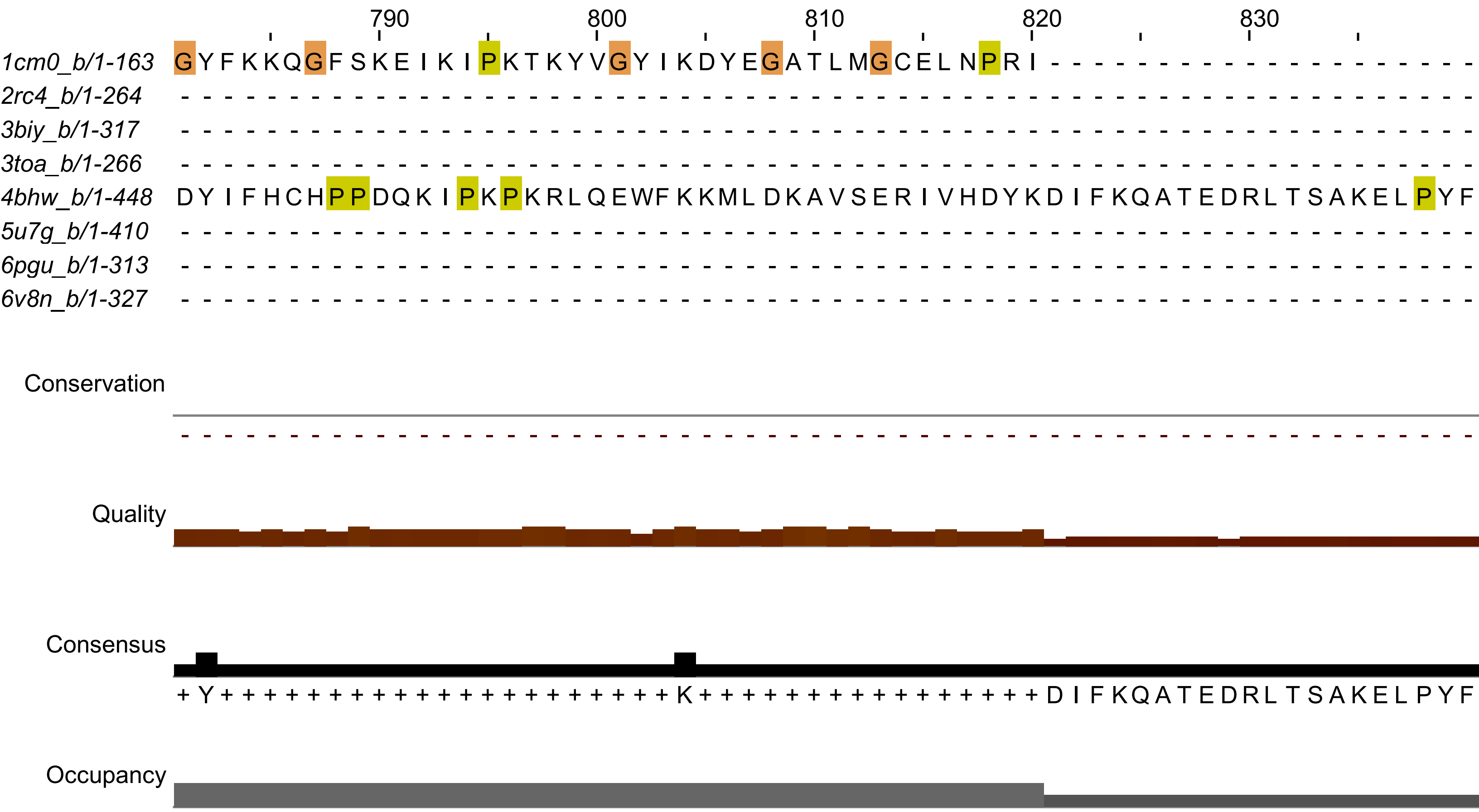
790
800
810
820
830
1cm0_b/1-163 GYFKKQGFSKEIKIPKTKYVGYIKDYEGATLMGCELNPRI--------------------
2rc4_b/1-264
3biy_b/1-317
3toa_b/1-266
4bhw_b/1-448 DYIFHCHPPDQKIPKPKRLQEWFKKMLDKAVSERIVHDYKDIFKQATEDRLTSAKELPYF
5u7g_b/1-410
6pgu_b/1-313
6v8n_b/1-327
Conservation
Quality
Consensus
+Y++++++++++++++++++++++K+++++++++++++++DIFKQATEDRLTSAKELPYF
Occupancy

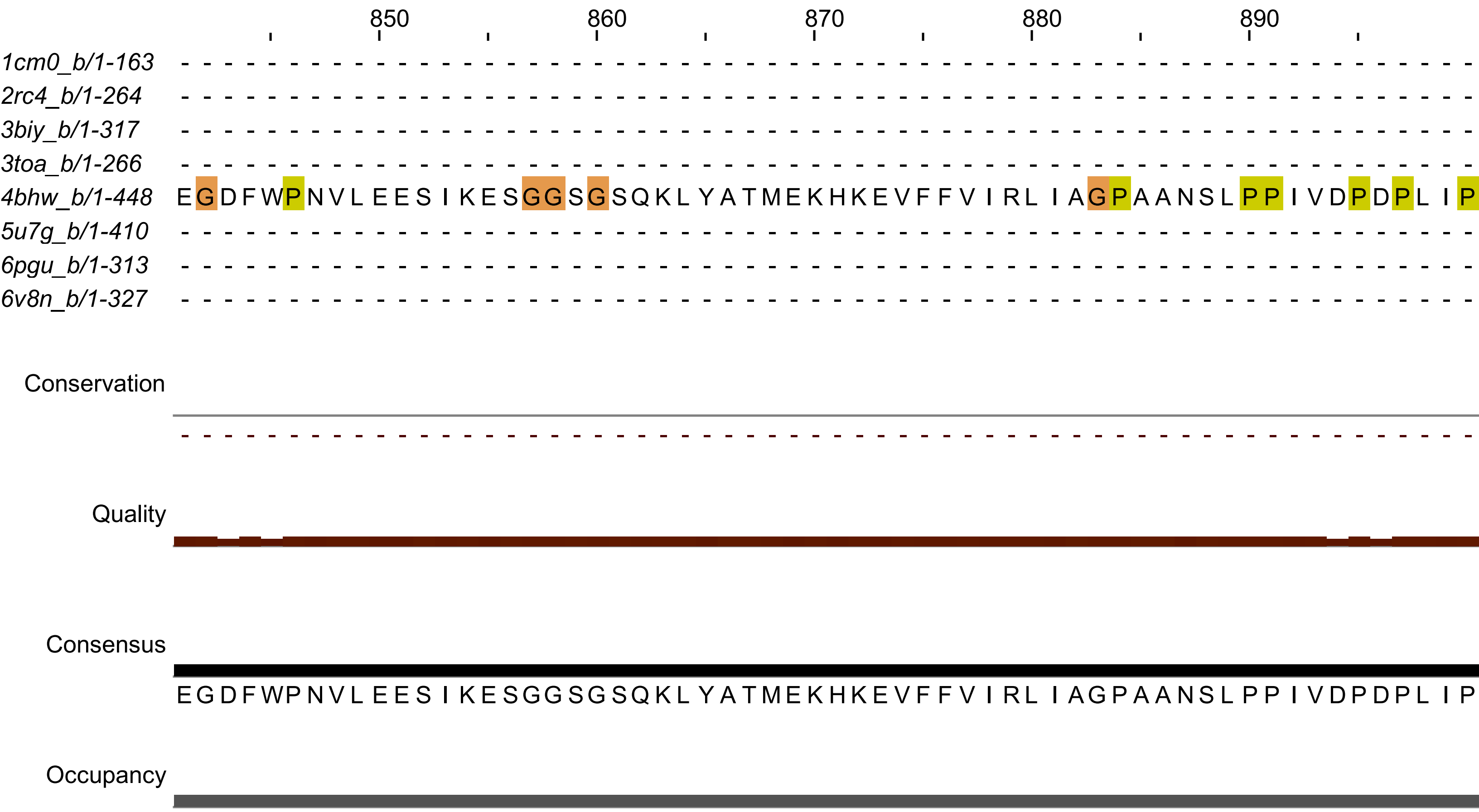

850
860
870
880
890
1cm0_b/1-163
2rc4_b/1-264
3biy_b/1-317
3toa_b/1-266
4bhw_b/1-448 EGDFWPNVLEESIKESGGSGSQKLYATMEKHKEVFFVIRLIAGPAANSLPPIVDPDPLIP
5u7g_b/1-410
6pgu_b/1-313
6v8n_b/1-327
Conservation
Quality
Consensus
EGDFWPNVLEESIKESGGSGSQKLYATMEKHKEVFFVIRLIAGPAANSLPPIVDPDPLIP
Occupancy

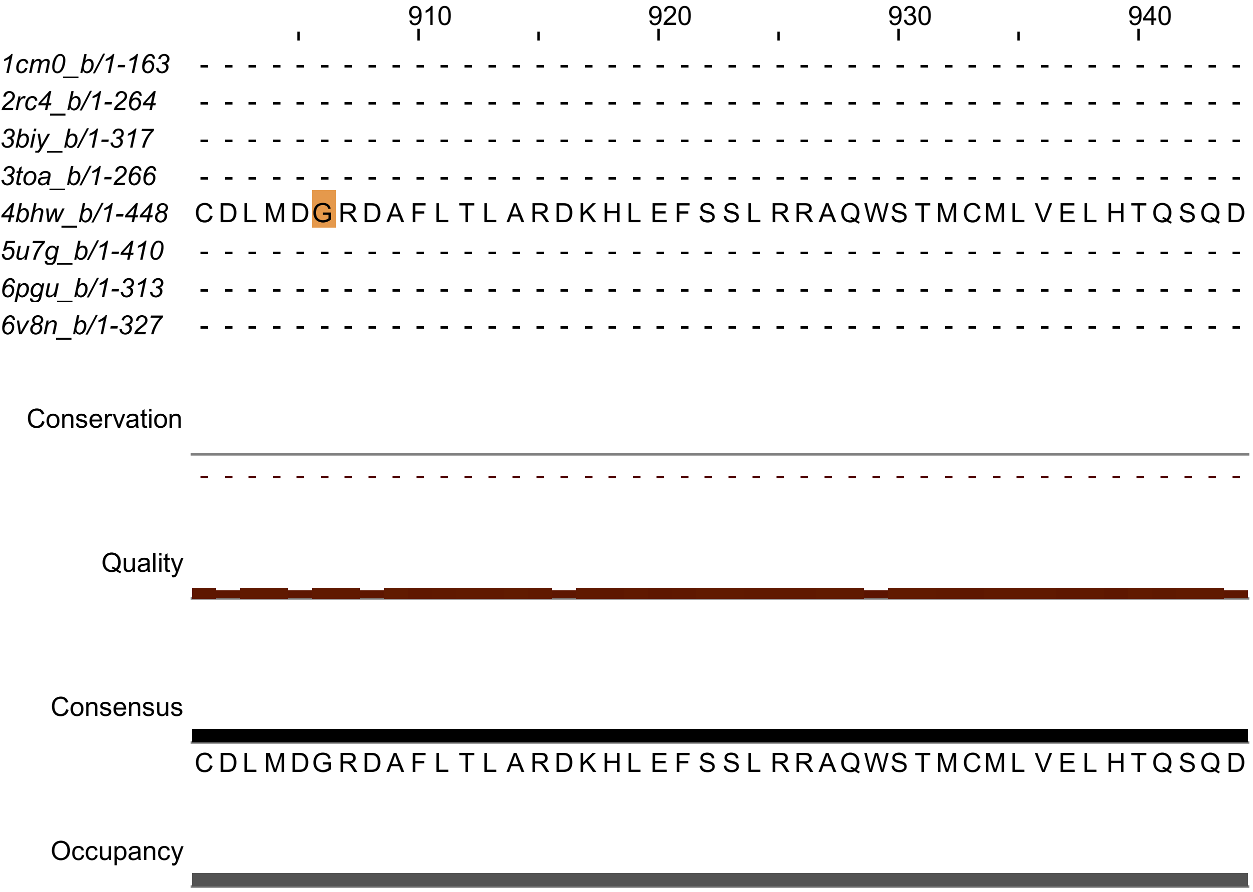
910
920
930
940
1cm0_b/1-163
2rc4_b/1-264
3biy_b/1-317
3toa_b/1-266
4bhw_b/1-448 CDLMDGRDAFLTLARDKHLEFSSLRRAQWSTMCMLVELHTQSQD
5u7g_b/1-410
6pgu_b/1-313
6v8n_b/1-327
Conservation
Quality
Consensus
CDLMDGRDAFLTLARDKHLEFSSLRRAQWSTMCMLVELHTQSQD
Occupancy
